# High gene expression of inflammatory markers and IL-17A correlates with severity of injection site reactions of Atlantic salmon vaccinated with oil-adjuvanted vaccines

**DOI:** 10.1186/1471-2164-11-336

**Published:** 2010-05-27

**Authors:** Stephen Mutoloki, Glenn A Cooper, Inderjit S Marjara, Ben F Koop, Øystein Evensen

**Affiliations:** 1 Department of Basic Sciences and Aquatic Medicine, Norwegian School of Veterinary Sciences, P.O. Box 8146 Dep. 0033 Oslo, Norway; 2 Centre for Biomedical Research, University of Victoria, PO Box 3020 STN CSC Victoria, B.C. V8W 3N5, Canada

## Abstract

**Background:**

Two decades after the introduction of oil-based vaccines in the control of bacterial and viral diseases in farmed salmonids, the mechanisms of induced side effects manifested as intra-abdominal granulomas remain unresolved. Side effects have been associated with generation of auto-antibodies and autoimmunity but the underlying profile of inflammatory and immune response has not been characterized. This study was undertaken with the aim to elucidate the inflammatory and immune mechanisms of granuloma formation at gene expression level associated with high and low side effect (granuloma) indices.

Groups of Atlantic salmon parr were injected intraperitoneally with oil-adjuvanted vaccines containing either high or low concentrations of *Aeromonas salmonicida *or *Moritella viscosa *antigens in order to induce polarized (severe and mild) granulomatous reactions. The established granulomatous reactions were confirmed by gross and histological methods at 3 months post vaccination when responses were known to have matured. The corresponding gene expression patterns in the head kidneys were profiled using salmonid cDNA microarrays followed by validation by real-time quantitative PCR (qPCR). qPCR was also used to examine the expression of additional genes known to be important in the adaptive immune response.

**Results:**

Granulomatous lesions were observed in all vaccinated fish. The presence of severe granulomas was associated with a profile of up-regulation of innate immunity-related genes such as complement factors C1q and C6, mannose binding protein, lysozyme C, C-type lectin receptor, CD209, Cathepsin D, CD63, LECT-2, CC chemokine and metallothionein. In addition, TGF-β (p = 0.001), IL-17A (p = 0.007) and its receptor (IL-17AR) (p = 0.009) representing T_H_17 were significantly up-regulated in the group with severe granulomas as were arginase and IgM. None of the genes directly reflective of T_H_1 T cell lineage (IFN-γ, CD4) or T_H_2 (GATA-3) responses were differentially expressed.

**Conclusions:**

Granulomatous reactions following vaccination with oil-based vaccines in Atlantic salmon have the profile of strong expression of genes related to innate immune responses. The expression of TGF-β, IL-17A and its receptor suggests an involvement of T_H_17 T cell lineage and is in conformity with strong infiltration of neutrophils and macrophages into inflamed areas. Arginase upregulation shows that macrophages in these reactions are alternatively activated, indicating also a T_H_2-profile. To what extent the expression of IL-17A and its receptor reflects an autoimmune vaccine-based reaction remains elusive but would be in conformity with previous observations of autoimmune reactions in salmon when vaccinated with oil-based vaccines.

## Background

In previous studies of concomitant histomorphological changes and antigen retention in Atlantic salmon (*Salmo salar *L.), we showed that intraperitoneal injection with oil-adjuvanted vaccines induces injection-site granulomas [1,2]. The mechanisms underlying the reactions are not well understood. In higher vertebrates, two polar forms of granulomas have traditionally been recognized [3,4]. The first type consists of organized nodular lesions comprising of epithelioid and multinucleate giant cells towards the center of the lesions, surrounded by fibrous tissue. Aggregates of lymphocytes are often present in the periphery. This type of granulomas is associated with a T_H_1 immune response and is dominated by interleukin 12 (IL-12), interferon-γ (IFN-γ), IL-2 and tumor necrosis factor alpha (TNF-α) resulting in macrophage activation and a strong cell-mediated immunity [5]. The second type is characterized by a lack of organization and is a T_H_2 type where IL-4, IL-5 and IL-13 dominate with activation of B cells, eosinophils, mast cells and a strong antibody production [6].

Recently, a third type of CD4 effector T cell lineage, T_H_17, has been discovered following the realization that mice deficient in IFN-γ or its receptor were not resistant to experimental autoimmune encephalomyelitis, an autoimmune disease previously associated with a T_H_1 response [7-11]. The T_H_17 effector cells are distinct from T_H_1 or T_H_2 types and have a cytokine profile predominated by IL-17A, IL-17F, IL-6, IL-21 and IL-22 (reviewed in ref. [12]). These T cells appear to function in the clearance of pathogens that have not been adequately handled by T_H_1 or T_H_2 cells but have also been implicated in several autoimmune diseases [13].

In mammals, the differentiation of naïve CD4 T helper cells into T_H_17 cells is induced by TGF-β in combination with other cytokines such as IL-6, IL-21 and IL-23 [14,15]. IL-21 also plays an auto-amplification role for T_H_17 cells while IL-23 is essential for stabilization [16,17]. IL-17 (IL-17A) is the hallmark of T_H_17 effector cells whose functions though not fully understood, includes the amplification of the immune response by the induction of other cytokines such as IL-6, TNFα, regulated on activation normal T cell expressed and secreted (RANTES) and monocyte chemoattractant protein-1 (MCP-1) [18,19]. Its capacity to cooperate with cytokines of the innate immunity to promote inflammation suggests that it is a bridge between innate and adaptive responses [20]. IL-17 binds to its receptor (IL-17AR) on target cells resulting in signaling via Act1 or CIKS, TNFR-associated factor 6 (TRAF6) and NF-κB and plays a role in inducing the recruitment of neutrophils and macrophages [21-23].

In line with the T_H_1/T_H_2 paradigm, the type of macrophage activation has been shown to parallel these responses [24]. In mice, activated macrophages metabolize arginine by two alternative pathways involving the enzymes inducible nitric oxide synthase (iNOS) or arginase [24]. Synthesis of reactive oxygen species (ROS) and NO from L-arginine by iNOS in the so-called classical pathway is associated with the T_H_1 response [25,26]. NO is a crucial host-protective, antimicrobial effector molecule as well as a potential host destructive mediator in diverse settings of immunopathology [27-29]. On the other hand, metabolism of arginine by arginase in the alternative pathway, converting L-arginine into ornithine and urea [24] is associated with the T_H_2 response. Macrophages in this state of activation play an important role against extracellular pathogens by showing increased phagocytic activity and enhanced gene expression of MHC class II. In addition, alternatively activated macrophages promote proliferation and antibody class switching and function in allergic reactions and wound healing processes [30]. Both iNOS and arginase compete for the common substrate L-arginine and the balance between the two enzymes is competitively regulated by T_H_1 and T_H_2 T helper cells via their secreted cytokines.

In Atlantic salmon, the presentation of granulomas following vaccination with oil-based vaccines is mainly in the form of diffuse granulomatous infiltration of inflammatory cells although in some cases, more organized lesions with lymphocyte infiltrations have been observed. There are, however, very few studies that address inflammatory or immunological reactions at genetic level. It is not certain whether T_H_1/T_H_2/T_H_17 responses exist in salmonids as in mammalian although many cytokines known to drive these responses including IFN-γ have been cloned, sequenced and characterized [31,32]. Interestingly, the different pathways of arginine metabolism in macrophages have been demonstrated [30,33-35] and more recently, vaccine-associated autoimmunity has also been implicated in Atlantic salmon [36].

Oil-adjuvanted vaccines act through several mechanisms such as the "depot effect" where antigens are retained at the injection site and released over extended periods of time; the enhancement of antigen presentation as well as antigen distribution or targeted immune activation [37]. In fish, antigens are retained in granulomas at the injection site [2]. Their distribution to head kidney and spleen following injection has been demonstrated [38]. The head kidney acts as a 'draining lymph node' for the peritoneal cavity in salmonids [39] and the retention of antigens for months in the head kidney [38,40] demonstrates that this organ plays a role in the immune responses.

In previous studies, we characterized the development of granulomatous reactions following vaccination with oil adjuvanted vaccines both grossly and histopathologically [2,41,42]. In these studies, we demonstrated that different antigens have different capacities to induce inflammation; and that the antigen-adjuvant combinations are responsible for most of the injection-site pathology. We also observed that the severity of inflammatory reactions increased with antigen concentration in the vaccines [41]. In the present study, we have taken advantage of this knowledge to develop skewed models of granulomatous reactions for profiling genes involved in inflammatory and immunological changes. The objective was to identify gene expression profiles associated with mild and severe granulomatous reactions by comparing the patterns in fish injected with standard vaccines inducing mild reactions on one side and those with severe reactions (injected with vaccines containing 6× the antigen concentration) on the other. Gross and histopathological examinations of the pyloric caeca (injection-site), as well as the gene expression profiles of both head kidney tissues and injection site of the same individuals were examined side by side at 3 months post vaccination (when local granulomas are well established [2]) using a salmonid cDNA microarray [43].

## Results

### Establishment of granulomatous reactions in different groups of fish

Gross lesions were observed at the injection sites of all fish and were characterized by varying degrees of adhesions with tiny fibrous strands between internal organs such as the pyloric caeca, pancreas and the abdominal wall. Skewed reactions were observed in fish vaccinated with *M. viscosa *vaccines, with mild reactions in FO-7 and severe ones in FO-8 (with a significant difference of p < 0.01) as demonstrated by the side effects scores (Table 1). By contrast, only mild reactions with minor differences were observed between FO-1 and FO-2 (*A. salmonicida*) groups. Melaninisation was present in the peritoneal cavities of fish in all groups albeit very mild (results not shown).

**Table 1:** Side effects and inflammatory scores.

Group	Side effect score*	Inflammatory score (μm2)
FO-1	1.5 (1-2)	50 (30)
FO-2	2 (1-2.5)	590 (340)
FO-7	2 (1-2.5)	490 (240)
FO-8	3 (2-4)	3820 (430)

*Side effects scores of Atlantic salmon following intraperitoneal injection with oil adjuvanted vaccines were graded on a scale of 0-6 and are given as median and range in brackets. Inflammatory scores are represented by means and standard errors in brackets. n = 15.

**Figure 1:**
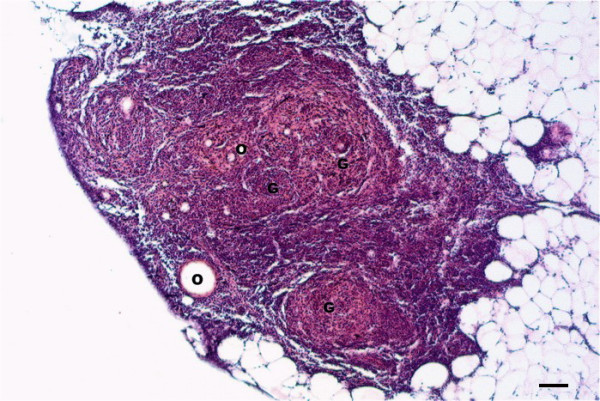
**Granulomas at injection-site of Atlantic salmon at 12 weeks following injection**. Well-circumscribed granulomas (G) induced by oil-adjuvanted vaccine containing standard amount of *Moritella viscosa *antigens. Note negative impression of oil droplets (O). Bar = 50 μm.

**Figure 2:**
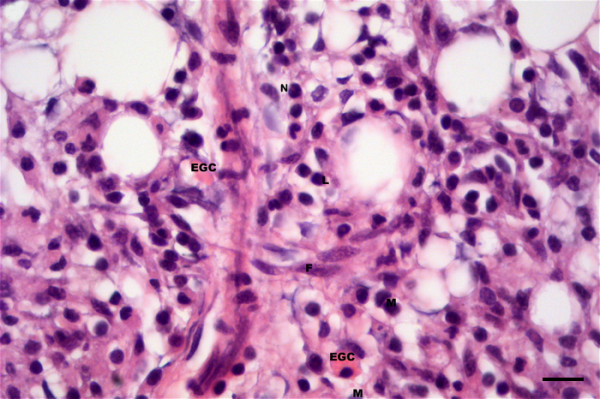
**Mixed cell infiltrate at injection-site of Atlantic salmon at 12 weeks following injection**. Cellular composition of inflammation consisting of macrophages (M), eosinophilic granular cells or mast cells (EGC), lymphocytes (L) and a few neutrophils (N). Fibroblasts are also distinct (F). Bar = 10 μm.

**Figure 3:**
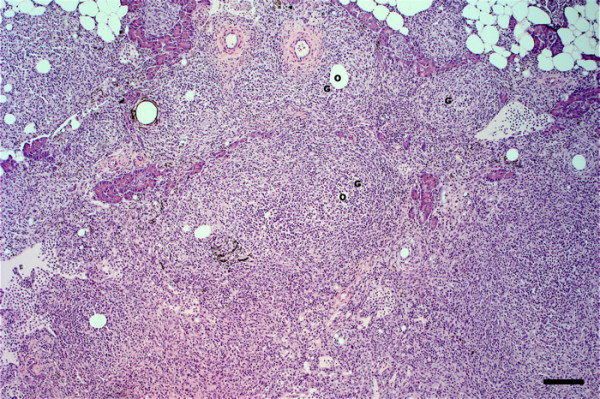
**Severe inflammatory reaction at injection-site of Atlantic salmon at 12 weeks following injection**. A severe granulomatous reaction with diffuse granulomas (G) induced by vaccine containing 6× standard *M. viscosa *antigens (FO-8). O- negative impression of oil droplets. Bar = 100 μm.

**Figure 4:**
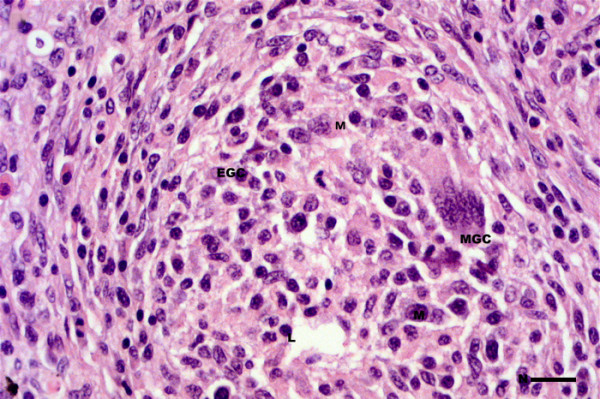
**Cellular composition of severe vaccine granuloma of Atlantic salmon at 12 weeks following injection**. Granulomas constitute of large macrophages (M), several lymphocytes (L), EGCs, and multinucleate cells (MGC). Bar = 15 μm.

Microscopically, granulomas in the form of well-circumscribed formations surrounding negative impressions of oil-droplets were observed between the blind sacs of the pyloric caeca, and between the pylorus and the pancreas (Figure 1). In mild reactions (FO1, FO-2 and FO-7) the granulomas were small and localized (Figure 1). The cellular composition consisted of epithelioid cells with fibroblastic reaction. Lymphocytes, macrophages, mast cells (eosinophilic granule cells) and a few neutrophils were also present (Figure 2). In severe reactions (FO-8), more granulomas were observed in each reaction (Figure 3). These reactions had similar cellular compositions as mild reactions but with higher numbers of neutrophils, macrophages and lymphocytes (Figure 4) being present in the granulomas, but with fewer mast cells.

### Microarray results

**Figure 5:**
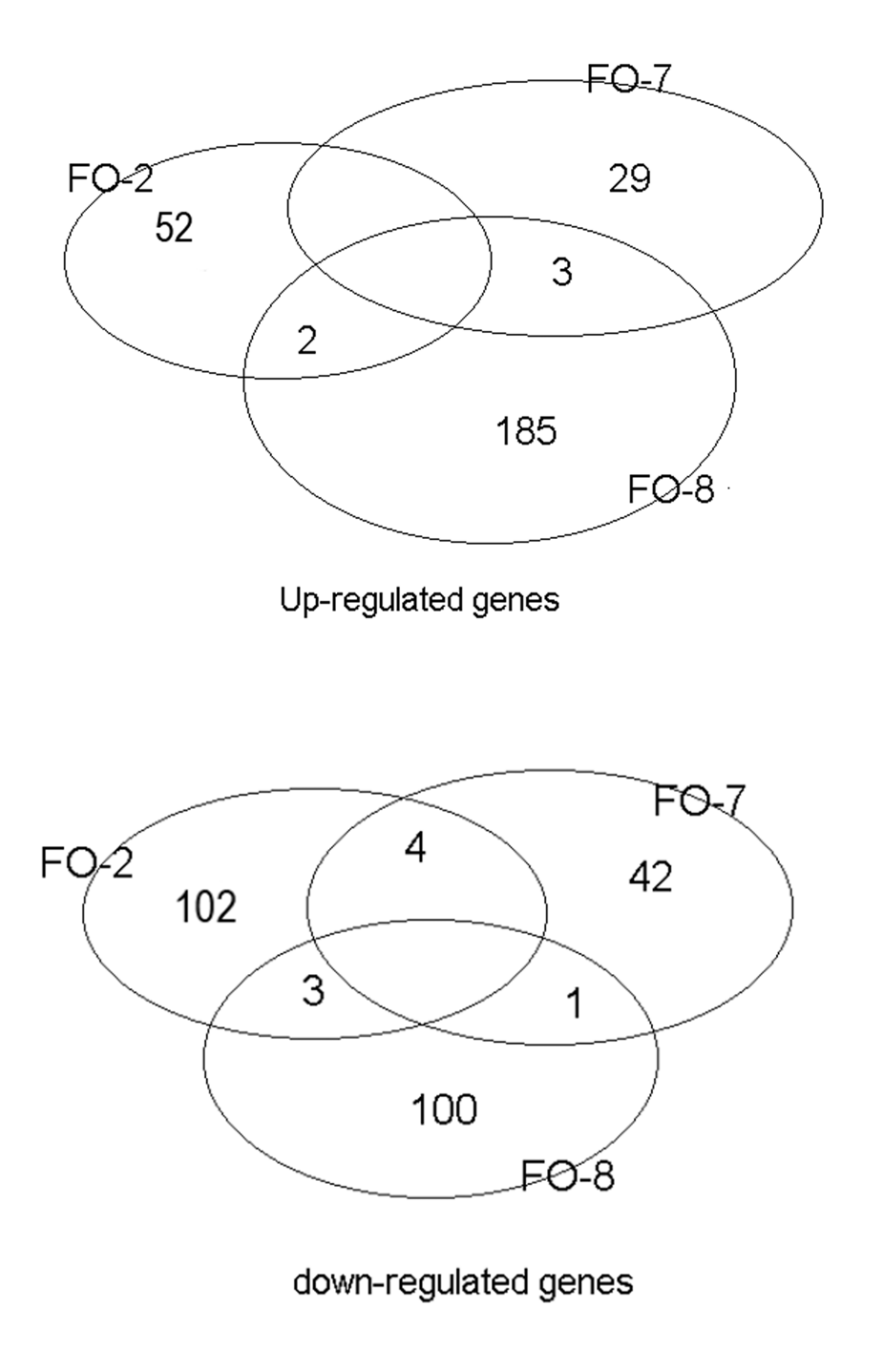
**Regulated genes in different groups of Atlantic salmon as determined by microarrays**. FO-2 = *A. salmonicida *(6 × standard concentration - sc); FO-7 = *M. viscosa *(sc); FO-8 = *M. viscosa *(6 × sc).

523 genes representing 3.3% of the genes spotted on the arrays were differentially expressed in all groups, with very few genes up- or down-regulated in more than one group (Figure 5). Differentially expressed genes were classified according to putative functional categories with the aid of Uniprot and NCBI database searches. The assignment of genes to groups was done preferentially to the most specific group. It should be noted however that most of the assignments were based on gene ontology as very few functional studies of these genes have been undertaken in Atlantic salmon.

Groups of differentially expressed genes perceived to be of particular interest in this study is listed in Additional file 1. There was a distinct pattern of gene expression in the head kidneys of fish with severe injection site reactions (FO-8) on one side and mild reactions (FO-2 and FO-7) on the other. These genes can be broadly categorized into innate and adaptive immunity. In the FO-8 group, several genes associated with innate immunity were up-regulated in the head kidneys including those encoding humoral defensive proteins such as complement factors and lysozyme C, the most highly expressed in this study (7.1 fold). Others were genes associated with antigen recognition and processing by phagocytes, antigen presentation, cellular signaling including CC chemokine and leukocyte cell derived chemotaxin 2 (LECT-2), matrix and cellular differentiation, anti-oxidative stress and inflammation. In contrast, very few of these genes were expressed in FO-7 or FO-2.

As for genes associated with adaptive immunity, clones of membrane-bound and soluble forms of IgM were up-regulated in FO-8 while several others associated with T lymphocytes such as T-cell receptor alpha chain, signaling lymphocyte activation molecule-associated protein, Tyrosine kinase ZAP-70 and Thymus-specific serine protease precursor were down regulated. Only one clone of IgM heavy chain was up-regulated in FO-7 while none were observed in FO-2.

As might be expected, a significant number of up-regulated genes were involved in transcription/translation and metabolism (18.8%). Some unknown genes (16.6%) were up-regulated, mostly in FO-8 while 22.6% were down-regulated, mostly in FO-2. 50.9% of the genes could not be clearly classified into inflammatory and immune responses (Additional file 2).

### Validation of microarray results by real-time qPCR

Twenty-six genes identified by microarrays as differentially regulated were validated by real-time qPCR. Their selection was based on their putative roles in granulomatous inflammation. A few additional genes were randomly selected and validated by real-time qPCR for representational purposes. In the FO-8 group, gene expression results by microarrays were confirmed by qPCR (88%). Lesser agreement was observed between the two platforms in FO-7 (54%) and FO-2 (61%) groups.

**Figure 6:**
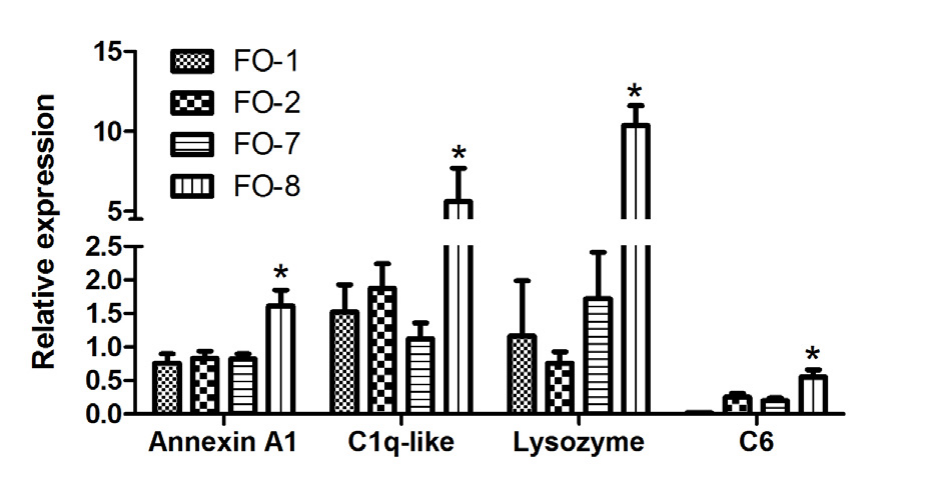
**Markers of inflammation expressed in the head kidney of Atlantic salmon injected with different oil-based vaccines**. Quantitative real-time RT-PCR (qPCR) expression of genes identified as upregulated by microarray. FO-1 = *A. salmonicida *(sc); FO-2 = *A. salmonicida *(6 × sc); FO-7 = *M. viscosa *(sc); FO-8 = *M. viscosa *(6 × sc). Gene expression ratios normalized to Elongation factor 1α (EF1α). Error bars = standard error (SE), n= 7. *p < 0.05.

**Figure 7:**
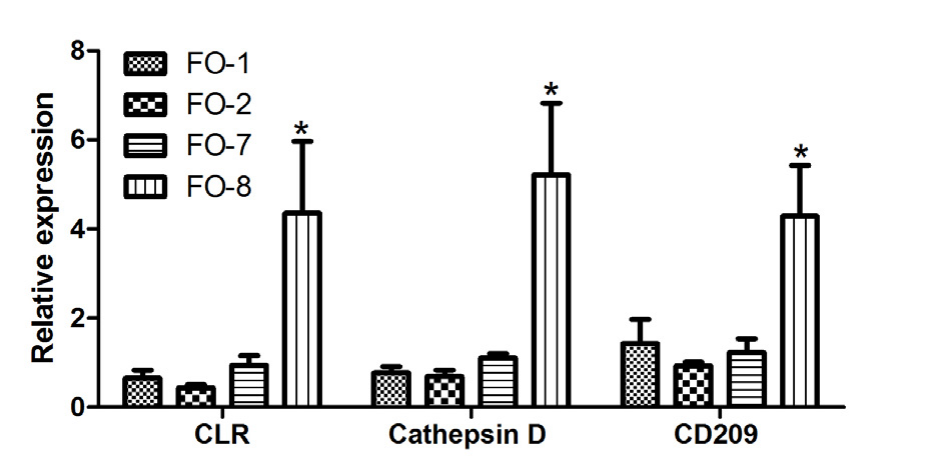
**Antigen processing and presentation genes expressed in the head kidney of Atlantic salmon injected with different oil-based vaccines**. qPCR expression studies of C-type lectin receptor (CLR), antigen recognition and processing by phagocytes (cathepsin D) and antigen presentation (CD209) genes identified as upregulated by microarray. FO-1 = *A. salmonicida *(sc); FO-2 = *A. salmonicida *(6 × sc); FO-7 = *M. viscosa *(sc); FO-8 = *M. viscosa *(6 × sc). Gene expression ratios normalized to EF1α. Error bars = SE, n= 7. *p < 0.05.

**Figure 8:**
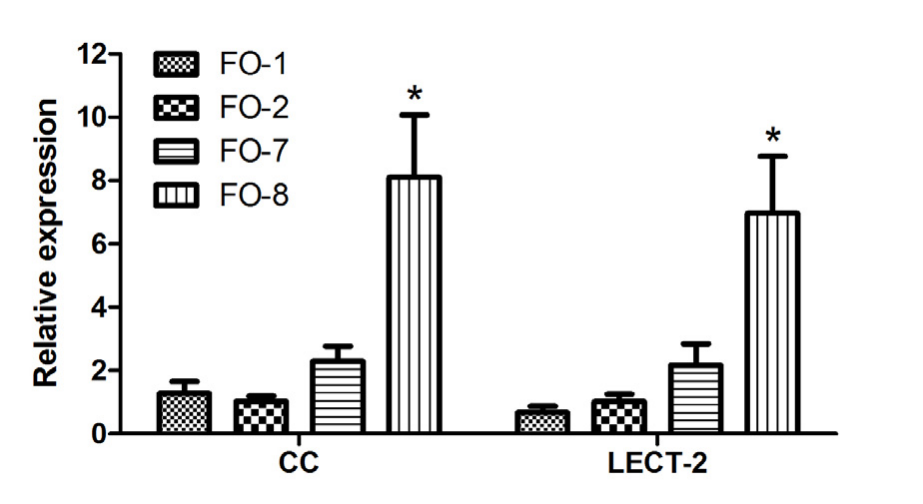
**Cellular signaling and chemotaxin markers expressed in the head kidney of Atlantic salmon injected with different oil-based vaccines**. qPCR expression studies of chemokine (CC) and leukocyte cell derived chemotaxin 2 (LECT 2) genes identified as upregulated by microarray. FO-1 = *A. salmonicida *(sc); FO-2 = *A. salmonicida *(6 × sc); FO-7 = *M. viscosa *(sc); FO-8 = *M. viscosa *(6 × sc). Gene expression ratios normalized to EF1α. Error bars = SE, n= 7. *p < 0.05.

**Figure 9:**
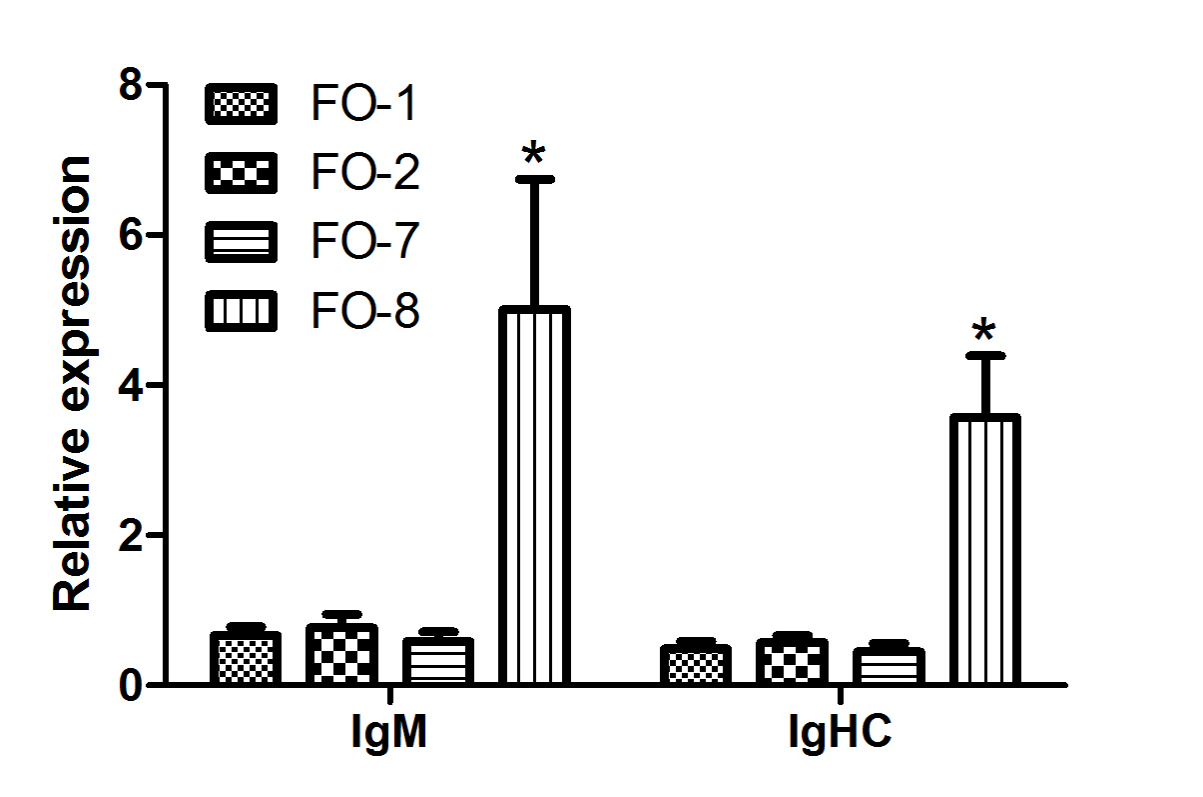
**Immunoglobulin genes expressed in the head kidney of Atlantic salmon injected with different oil-based vaccines**. qPCR expression studies of IgM and immunoglobulin heavy chain constant region (IGHC) genes identified as upregulated by microarray. FO-1 = *A. salmonicida *(sc); FO-2 = *A. salmonicida *(6 × sc); FO-7 = *M. viscosa *(sc); FO-8 = *M. viscosa *(6 × sc). Gene expression ratios normalized to EF1α. Error bars = SE, n= 7. *p < 0.05.

qPCR results for individual genes like Annexin A1, C1q-like, lysozyme and C6 were found significantly higher expressed in FO8 groups compared to the others (Figure 6). Similarly genes associated with antigen recognition and processing by phagocytes (cathepsin D), antigen presentation (CD209) (Figure 7), cellular signaling including CC chemokine and leukocyte cell derived chemotaxin 2 (LECT-2) were similarly upregulated (Figure 8). Both membrane and secreted forms of IgM were verifiably up-regulated in FO-8 compared to other groups (Figure 9). Some T cell related genes such as lymphocyte cytosolic protein 1 and signaling lymphocyte activation molecule, though down-regulated in microarrays, were not differentially expressed (not shown).

### Profiling of inflammatory and immune genes

**Figure 10:**
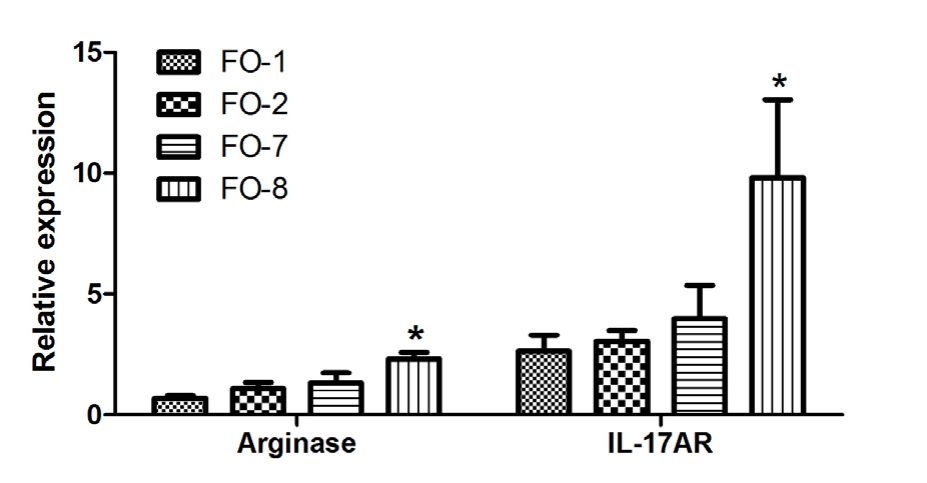
**Arginase and IL17AR genes expressed in the head kidney of Atlantic salmon injected with different oil-based vaccines**. qPCR expression studies of arginase and IL17A receptor (IL17AR) identified as upregulated by microarray. FO-1 = *A. salmonicida *(sc); FO-2 = *A. salmonicida *(6 × sc); FO-7 = *M. viscosa *(sc); FO-8 = *M. viscosa *(6 × sc). Gene expression ratios normalized to EF1α. Error bars = SE, n= 7. *p < 0.05.

**Figure 11:**
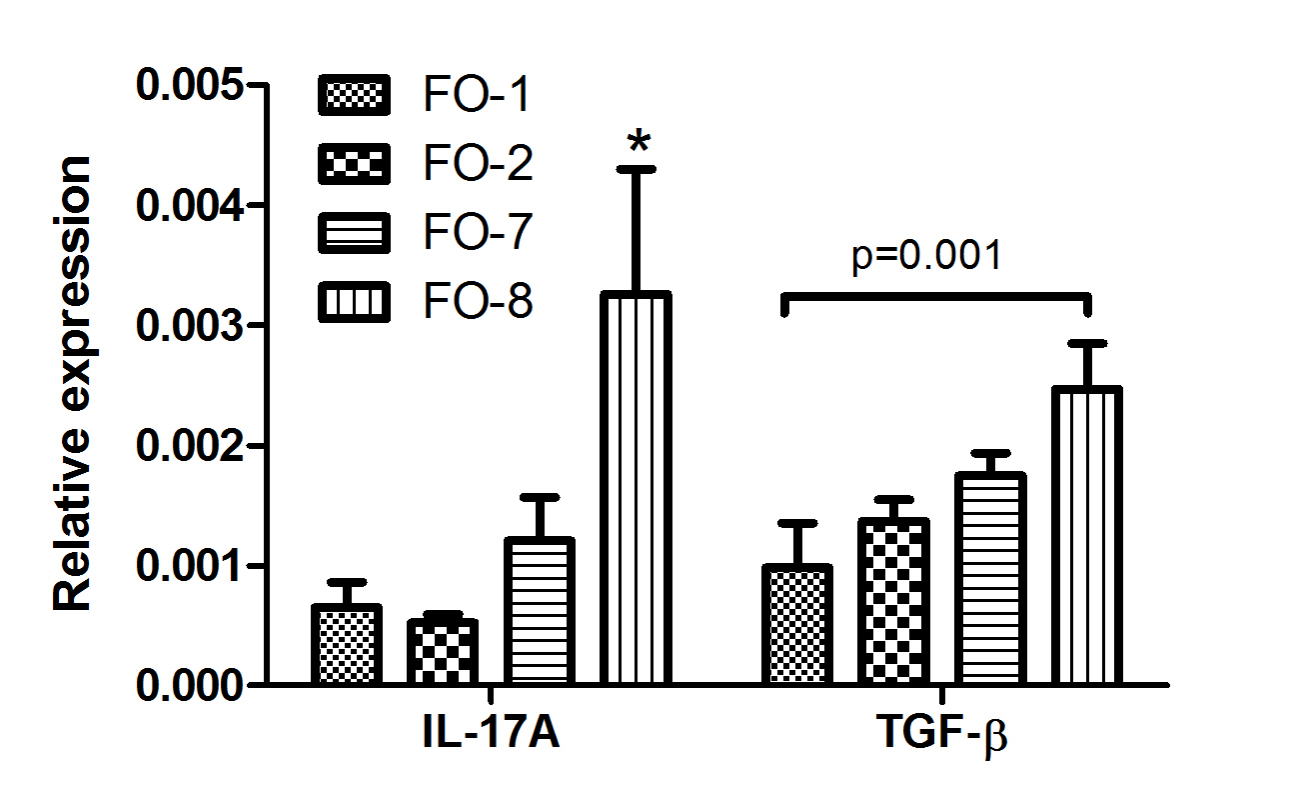
**Cytokine genes expressed in the head kidney of Atlantic salmon injected with different oil-based vaccines**. qPCR expression studies of IL17A and transforming growth factor β (TGF-β) genes identified as upregulated by microarray. FO-1 = *A. salmonicida *(sc); FO-2 = *A. salmonicida *(6 × sc); FO-7 = *M. viscosa *(sc); FO-8 = *M. viscosa *(6 × sc). Gene expression ratios normalized to EF1α. Error bars = SE, n= 7. *p < 0.05.

Arginase 1 (p < 0.05), IL-17A (p = 0.007) and IL-17A-receptor (AR) (p = 0.009) were significantly up-regulated in FO8 compared to all other groups (Figures 10 and 11). TGF-β was significantly up-regulated in FO-8 compared to FO-1 (p = 0.001), with a similar general trend in the strength of expression in different groups as the severity of lesions (Figure 11, Table 1). No difference was observed in the expressions of the genes encoding IFN-γ, CD4, CD8, IL-6 receptor, IL-10, iNOS, GATA-3 and Granzyme A between groups (not shown).

## Discussion and conclusions

Several genes including complement factors C1q and C6, mannose binding protein, lysozyme C, C-type lectin receptor, CD209, Cathepsin D, CD63, LECT-2, CC chemokine and IgM were identified in the head kidney as associated with severe injection-site granulomatous reactions in this study. The expression of these genes corresponds with gene profiles of an active inflammation [44-47] and corroborate previous reports that vaccine-based granulomas are associated with a chronic, active inflammation [2]. Furthermore, the finding that IL-17A and its receptor (IL-17AR) representing T_H_17 cells were up-regulated in fish with severe reactions while none of the genes directly reflective of T_H_1 T cell lineage (IFN-γ, CD4) or T_H_2 (GATA-3) differentiation were differentially expressed is interesting in light of the involvement of the T_H_17 cells in autoimmune responses [13].

Chronic, active inflammation is consistent with the presence of neutrophils and macrophages at the injection site of FO-8. In this group, genes encoding LECT-2 and CC chemokine were verifiably up-regulated in head kidney indicating an active inflammatory process in the "draining lymph node." Further to this, the up-regulation of mRNA transcripts of complement factors C1q and C6, mannose binding protein, lysozyme C, innate cell-associated proteins of the C-type lectin receptors, CD209, Cathepsin D and CD63, anti-oxidative genes such as metallothionein and oxidative stress-related genes are additional indications of active inflammatory processes [48] in fish with severe granulomatous reactions.

Another intriguing result was the finding that *M. viscosa *(FO-7&8) is more pro-inflammatory than *A. salmonicida *(FO-1&2). In natural infection, lethality of these pathogens is in the reverse [49]. The cause of the difference in this study is not clear but it is not unlikely that the structure or quantity of pathogen-associated molecular patterns (PAMPs) [50], or even the orientation/presentation of antigens on the surface of oil droplets may be responsible. The importance of the different factors can only be solved in future studies.

Macrophages are the main cell type responsible for the uptake, breakdown, removal or sequestration of antigens. For an effective response, appropriate activation is necessary. The observed expression of arginase in this study is consistent with macrophages activated through the alternative pathway and is indicative of a T_H_2 response [51]. In humans, it has been documented that T_H_2 cytokines bias responses towards inadequate activation of macrophages resulting in less efficient control of *Mycobacterium leprae *infections and high bacterial counts inside granulomas leading to diffuse spreading of the infection [52]. To what extent macrophage activation plays a role in clearing of the vaccine antigens in salmonids is not known in detail. It is not possible to deduce from the results of this study whether the alternative macrophage activation pathway is an appropriate response but this should be a subject of further investigation.

The massive up-regulation of IgM mRNA transcripts in the head kidneys of fish with severe injection-site reactions (Additional file 1) is suggestive of a strong humoral response, another indication of a T_H_2 response consistent with reports of others [53,54]. It must be noted however that no tests to examine the specificity of antibodies were conducted in this study. It is thus unknown whether the bulk of the IgM mRNA transcripts detected in this study are indications of responses against vaccine-related antigens or if they were directed at self (autoimmune) antigens. Autoimmunity has previously been suggested as one of the etiologies of vaccine associated side effects in Atlantic salmon [36].

In fish with mild injection-site reactions, microscopic examinations revealed a process of wound healing. Mast cells and macrophages were observed in abundance at the injection site of fish in these groups (FO-1, FO-2 and FO-7). These cells have previously been associated with granulomatous reactions [2,55] and wound healing [42], although they have also been associated with the initiation of inflammation in Atlantic salmon [56]. In higher vertebrates T_H_2 responses have been shown to play an important role in wound healing [57] with mast cells producing or influencing the production of IL-4 and IL-10 tipping the response towards T_H_2 [58,59]. In the present study, no significant differences in the expression of GATA-3 or IL-10 were observed between different groups. Similarly, no differences were observed for IL-12, IFN-γ, CD4, CD8 and Granzyme A. As similar antigen preparations per pair of vaccines were used differing only in concentrations, the anticipation is that similar T_H _responses drove the inflammatory responses in all groups; the difference in severity being motivated by antigen concentration and determined mainly by the innate response.

Interestingly, the expression of IL-17A and its receptor showed similar trends as the severity of inflammatory profiles (Figures 10 and 11, and Table 1), being significantly up-regulated (p = 0.007 and 0.009, respectively) only in the group with severe granulomas. IL-17A is a pro-inflammatory cytokine produced predominantly by activated T cells. It acts through its receptor IL-17AR which, although ubiquitously expressed, its main responses in higher vertebrates are found in epithelial cells, endothelial cells, fibroblasts, macrophages and dendritic cells (reviewed in ref. [60,61]). It is an essential component of the IL-17A signaling whose induction is required for effective responses [60,62-64]. It has also been demonstrated to be up-regulated together with IL-17A in autoimmune diseases [65-67]. IL-17A expression induces the production of an array of cytokines and metalloproteases by fibroblasts, endothelial cells, macrophages and epithelia cells resulting in the induction of inflammation and recruitment of neutrophils [68]. These findings suggest that IL-17A/IL-17AR and therefore the T_H_17 response contributes to the induction of severe side effects in line with the higher number of infiltrating neutrophils and macrophages in this group. To what extent autoimmunity as previously reported [36] is involved in these responses remains elusive as the presence of autoantibodies was not assessed in this study.

The involvement of TGF-β in severe granulomatous reaction in this study is compelling given the expression patterns exhibited in different groups (Figure 11) relative to the degree of side effect lesions (Table 1). In mammals, the expression of TGF-β has previously been associated with the recruitment of polymorphonuclear cells to inflammatory sites [69]. Together with IL-6, IL-21 or IL-23, it has also been demonstrated to be essential in the commitment of naïve T cells into T_H_17 cells [14,15]. TGF-β is produced by cells of the innate immune system [70] as well as regulatory T cells (T-regs) [71,72]. Although only the receptor for IL-6 and not the cytokine itself was examined in the present study and found not to be induced, it is not unlikely that a different expression pattern would have been observed with the latter. On the other hand, this finding may suggest that other cytokines such as IL-21 or IL-23 act in concert with TGF-β to regulate T_H_17 cells in the induction of severe granulomatous reactions in Atlantic salmon.

The screening of differentially expressed genes for granulomatous reactions using microarrays in this study was done by examining head kidney tissues rather than the injection site. It is conceivable that more inflammatory genes would be up- or down-regulated at the latter compared to the former, as most inflammatory genes are known to act locally. In i.p. vaccinated fish, the injection site is the abdominal cavity and vaccine components localize primarily at the pyloric caeca/pancreas area [2,41]. Because of the anatomical co-localization of the pylorus with the pancreas, collection of homogeneous tissue for gene expression studies is problematic. An alternative approach would be to vaccinate the fish intramuscularly and examine gene expression at the injection site as previously reported [73]. However, for vaccines routinely administered i.p., this approach would have to be weighed against the prospect of modifying the immune response since different routes of vaccination are known to influence the resulting T_H _profiles [74].

In conclusion, the profile of the immune responses as assessed by gene expression to oil-adjuvanted vaccines in Atlantic salmon is strongly influenced by antigen content and also the type of antigens included in the vaccine preparation. There is a variation in the response along an axis of a chronic, active inflammation on one end to mild inflammation and wound healing on the other. Gene expression patterns indicative of neutrophil persistence and macrophage activation are biased towards a mixed reaction between T_H_2 and T_H_17 profiles in the head kidney.

## Methods

### Fish

About 250 Atlantic salmon (*Salmo salar *L; Bolax strain) weighing approximately 42 g each were procured from Lindås Fiskeoppdrett AS, Vågseidet in Norway. The fish were reared in continuously running fresh water tanks at Stiftelsen Industrilaboratoriet Høyteknologisenteret (ILAB) in Bergen, Norway. The water temperature was 17°C on average and the fish were fed on commercial dry pellets (EWOS Innovation, Bergen, Norway).

### Vaccines

Injection preparations used in this study are shown in Table 2. All preparations were bivalent experimental vaccines (with equal concentrations of infectious pancreatic necrosis virus in all vaccines as second antigens) from PHARMAQ AS, Overhalla, Norway, produced according to in-house standard procedures. The oil-based antigen preparations were formulated as water-in-oil (w/o), where the water phase (containing bacterial antigens) was dispersed into an oil phase (continuous phase containing emulsifiers and stabilizers). Emulsification of the antigens with adjuvant was done using a homogenizer with a standard emulsification stator/rotor connected to an emulsior screen. The potency of the standard vaccines was according to specifications given in European Pharmacopoeia monographs for *Aeromonas salmonicida*, RPS > 80 and for *Moritella viscosa *the potency was RPS > 60 and 60% control mortality in the non-vaccinated (according to internal standards of PHARMAQ). Sterility, free formaldehyde, inactivation, stress, viscosity and droplet size tests were all performed and standardized on blended bacterin according to standard procedures in the laboratory.

**Table 2:** Vaccine preparations used in the present study.

	Antigens	
	
Vaccine*	*Aeromonas salmonicida*	*Moritella viscosa*
FO-1	Standard concentration	0
FO-2	6 × standard concentration	0
FO-7	0	Standard concentration
FO-8	0	6 × standard concentration

*All vaccines were adjuvanted with mineral oil and were formulated as water-in-oil emulsions.

### Randomisation, marking and vaccination of the fish

The selection of fish was done by dip-netting followed by sequential allocation into groups. Four groups comprising 50 fish each were included in the present experiment. Marking was done by fin clipping. Prior to marking, the fish were anaesthetised using Chlorobutanol (Sigma, Steinheim, Germany) dissolved in rearing water at a concentration of 0.5 ml/L. The fish were vaccinated by injecting 0.1 ml of vaccine intraperitoneally (i.p.) through the ventral midline, about 1 to 1.5 pelvic fin lengths anterior to the pelvic fin base. Self-refilling syringes (Socorex, Ecublens, Switzerland) with 0.6 × 3 mm needles (Unimed, Lausanne, Switzerland) were used. After immunization, all the fish were placed into two fiberglass tanks, each with a water capacity of 500 L. Post vaccination mortality and appetite of the fish were monitored daily.

### Sample collection and processing

Sampling was done at 12 weeks post vaccination (p.v.). Fifteen fish from each group were randomly selected by dip netting, anaesthetized and then sorted into groups. Sampling was done as previously described [2]. Briefly, the fish were stunned by a blow to the head and the gill vein severed. A sample (max volume = 10 × 10 × 5 mm) of pyloric caeca and surrounding tissues (designated as the injection site) and head kidney were collected from each fish and stored in 10% phosphate buffered formalin for a minimum of 4 days. Thereafter, the samples were processed according to standard procedures employed for hematoxylin and eosin (H&E) staining. In addition, parallel samples of the head kidney and pyloric caeca were collected from individual fish and stored separately in RNA*later*^® ^(QIAGEN, Hilden, Germany) according to the manufacturer's instructions until required.

### Assessment of gross and microscopic lesions

Gross lesions in the peritoneal cavity of the fish were graded on an arbitrary scale of 0-6, where 0 = no lesions and 6 = severe as previously described [75]. For microscopic lesions, the size of inflammatory reaction of each fish was assessed by calculating the area of the inflamed lesions using computer-assisted microscopy on H&E stained sections as previously described [41]. Briefly, well-calibrated microscopical images were acquired at 100 × magnification by using a computer-controlled digital camera mounted on a light microscope. The circumferences of inflamed areas in digital images were marked manually. The number of pixels within the marked region was then converted to area with the help of the Image-Pro^® ^Analysis program (Media Cybernetics, L.P., Silver Spring, USA).

Data on gross lesions was subjected to Fisher's exact test while inflammatory reactions between groups were analysed by linear regression as previously described [42].

### Microarray-based screening of gene transcripts in head kidney tissues

Head kidney tissues, rather than those of the injection site (pylorus/pancreas) were used for the screening of gene transcripts by microarrays in this study due to the difficulty in obtaining homogeneity in the samples of the latter as the organs (pyloric caeca and pancreas) are anatomically intertwined. Because of this it was perceived that using injection site tissues would not give representative gene expression results.

### RNA isolation

RNA from head kidney tissues stored in RNA*later *was isolated by using the RNeasy^® ^minikit (QIAGEN, Hilden, Germany). Each tissue was homogenized by subjecting it to a mixer mill MM301 (Retsch GmbH & Co., Haan, Germany) for 2 min at 20 Hz in buffer RLT followed by total RNA extraction with on-column DNAse treatment according to the manufacturer's protocol to minimize DNA contamination. The quantification of RNA, purity and integrity assessment were done by using a NanoDrop ND-1000 spectrophotometer (NanoDrop technologies, Inc., Wilmington, USA) and the Agilent 2100 Bioanalyzer (Agilent technologies).

### Microarray study design and target preparation

**Figure 12:**
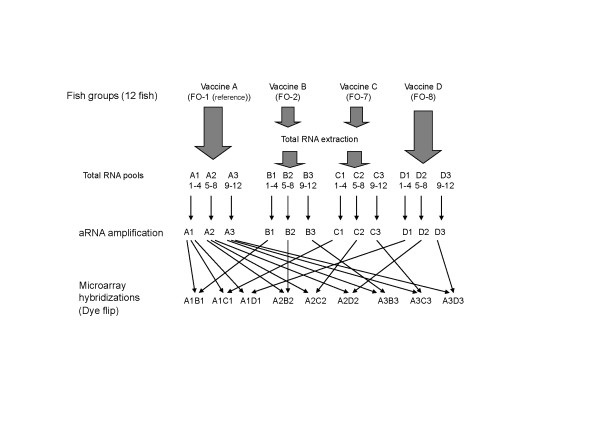
**Microarray study design**. Total RNA from 12 fish in each group was subdivided into pools of 4 fish each and subsequently used for hybridization. Vaccine A represents the reference group (FO-1 *A. salmonicida *standard concentration).

Fish were grouped according to the vaccines administered (Table 2). The group with the least gross and inflammatory changes (FO-1) was used as the reference in microarray experiments. Out of 15 fish sampled in each group, only total RNA from 12 fish, chosen on the basis of abundance and quality/integrity, was used for downstream analysis (Figure 12).

### RNA amplification

In order to generate sufficient amounts of targets for hybridization, each total RNA pool was subjected to one round of amplification using the Amino Ally MessageAMP™ II aRNA amplification kit (Ambion, Austin, USA) starting with 2 μg. Briefly, reverse transcription (RT) to synthesize the first cDNA strand was done using oligo(dT) primers containing a T7 promoter sequence. The RNA and primer mix was first denatured at 70°C for 10 min prior to the addition of the RT master mix. The first strand synthesis reaction was incubated at 42°C for 2 hrs. The second cDNA strand was synthesized by the addition of the second strand master mix and incubated at 16°C for 2 hrs. The resulting double stranded cDNA was purified by using column-based reagents, according to the protocol supplied with the kit. Finally, amino allyl-modified anti-sense RNA (aRNA) was synthesized by the addition of an *in-vitro *transcription master mix containing a 1:1 ratio of UTP to amino allyl-modified UTP and held at a temperature of 37°C for 14 hrs. The aRNA was then purified and stored at -80°C until required.

Coupling of aRNA was done with either Cy3 or Cy5 dye according to the Amino Ally MessageAMP™ II aRNA Amplification Kit protocol with minor modifications. Briefly, one vial of Cy3 or Cy5 monofunctional reactive dyes (Amersham, Buckinghamshire, UK) was resuspended in 22 μl of DMSO prior to coupling. 3 μg of aRNA was dried to completion in a vacuum centrifuge (Savant Instruments, Inc., Farmingdale, NY). The aRNA was resuspended in 4.5 μl of coupling buffer. Thereafter, 5.5 μl of dye was added to the aRNA:coupling buffer and incubated for 30 min in the dark. 2.25 μl of 4 M hydroxylalamine was then added to quench the reaction followed by 12.75 μl of water to obtain a total volume to 30 μl. Dye labeled aRNA purification was done according to the manufacturer's instructions.

### Array hybridization, scanning and analysis

The fabrication of the salmonid microarrays used in the present study is described elsewhere [43]. A complete description and list of the probes used on the arrays is available at http://web.uvic.ca/grasp.

The experiments were designed to comply with MIAME guidelines [76]. Three biological and 2 technical replicates (dye-swapped) were utilized in different combinations to hybridize to 18 arrays (Figure 12). The assignment of microarrays to treatment groups for hybridization was done randomly by using a random number generator. To minimize technical variability, target synthesis and hybridizations were done in batches of 6 where all treatment groups were equally represented.

Post-print processing of arrays was done by washing 2 × 5 min in 0.2% SDS, 5 × 1 min in MilliQ water followed by immersion in MilliQ water at 100°C for 3 min. The slides were dried by centrifugation at 512 × g for 5 min.

Array were prehybridized by placing them in (5 × SSC, 1% SDS and 3% BSA (Sigma, Steinheim, Germany)) and incubating at 49°C in a water bath for 11/2 hrs. Thereafter, the arrays were washed thrice for 20 sec in MilliQ water and dried by centrifugation as above. For hybridization, 500 ng of labeled target reconstituted to a final volume of 26 μl, 30 μL of 2 × formamide hybridization buffer (Genisphere, Hartfield, USA) and 4 μl LNT dT blocker were used. Hybridization was done at 49°C in a water bath for 16 hrs. The arrays were washed first in 2 × SSC, 0.1% SDS for 10 min at 49°C, 2 × 5 min in 2 × SSC, 0.1% SDS at room temperature, 2 × 5 min in 1 × SSC, 4 × 5 min in 0.1 × SSC followed immediately by centrifugation at 512 × g for 5 min.

Imaging was done at 10 μm resolution using ScanArray™ Express microarray scanner (Packard Bioscience). The Cy3 and Cy5 cyanine fluors were excited at 543 nm and 633 nm, respectively, at the same laser power (90%), with adjusted photomultiplier tube settings between slides to balance the Cy3 and Cy5 channels. Image analysis and fluorescent intensity data was extracted from Tiff images using ImaGene™ 5.6 Standard Edition software.

Data analysis (correction of background, setting of background corrected values < 0.01 to 0.01), normalization (Lowess) and analysis (formation and manipulation of fold change transcript lists) were performed in GeneSpring Gx (Agilent Technology). Clones were assigned as either up or down regulated based on two conditions: a ratio of at least 1.5 fold change in at least 3 of the six replicates and also a statistical p value of < 0.05. The data is deposited at NCBI's GEO repository under accession number **GSE8826 **and Platform **GPL2716**.

### Validation of microarray results by quantitative RT-PCR

The results obtained by microarray experiments were verified by real-time quantitative PCR (qPCR) by using the LightCycler 2.0 instrument and LightCycler^® ^FastStart DNA Master^PLUS ^SYBR Green I (Roche Diagnostics GmbH, Manheim, Germany) as previously described [77].

One microgram of total RNA from the head kidney or pyloric caeca of individual subjects was reverse transcribed using oligo (dT)_20 _and Superscript III Reverse transcriptase (Invitrogen, Carlsbad, CA, USA) according to the manufacturer's instructions. Thereafter, cDNA was scaled up to 100 μl with water. Two microlitres of the diluted cDNA was used as template for each qPCR reaction.

The primers employed in the quantification of PCR reactions are presented in Table 3. Designing of the primers was done using Primer3 software (Whitehead Institute of Biomedical Research, MA., USA) while primers were procured from MWG Biotech AG, Germany. The generation of a single product of the expected size for each assay was confirmed by electrophoresis of the PCR (ordinary) and also LightCycler q-PCR product on a 2% agarose gel. Quantitative PCR was done in duplicate and the melting curve analysis was applied to confirm the formation of a single product.

**Table 3:** Primers used for real-time quantitative PCR.

**Accession no**.	Name	Direction	Sequence	T*_A_	P** size (bp)
**CB510337**	T cell receptor alpha chain (TCAC)	Fwd	GCCTGGCTACAGATTTCAGC	66	107
		Rev	GGCAACCTGGCTGTAGTAGC		

**CA052383**	Complement component C6	Fwd	TCCAACGTGCCACTCTCCTC	64	131
		Rev	CCGAACAGGGCTTCTACACC		

**CA044561**	L-plastin (Lymphocyte cytosolic protein 1)	Fwd	CATGCGAACACAGTCAGAACC	62	111
		Rev	CAACGCCAAGTACGCTATCAC		

**CA059313**	Annexin A5	Fwd	GAAACTTCAACGCCAACCAAG	60	115
		Rev	TGTCTCTGGCTGTTGCTACG		

**CA062753**	SH2 domain protein 1A	Fwd	GGGAGTAGTCTCTGCTGTCC	62	95
		Rev	GGCTCTACTGCCTCTGTGTG		

**CB493440**	Proteasome subunit alpha type 4	Fwd	GTTCTAACCAATGAGCTGAGG	60	89
		Rev	AACGCTGTCACCAACTGCTC		

**CB511680**	Lysozyme C	Fwd	CACCGACTATGGCATCTTCC	58	129
		Rev	CTGACCGCCACTGTGATGTC		

**CA767935**	Cathepsin D	Fwd	CAGGCTGGTAAGACCATCTGC	58	127
		Rev	TGTTGTCACGGTCGAACACAG		

**DN048269**	Complement C3a	Fwd	GAGGAAAGGTGAGCCAGATG	58	106
		Rev	TGTGTGTGTCGTCAGCTTCG		

**CA043655**	Interferon regulatory factor-1 (IRF-1)	Fwd	GATGGGACCTGAACAAGGATG	58	132
		Rev	GAAGGGAGTTCATTGCACAGC		

**A064221**	MHC-II alpha	Fwd	GAACACAGCAGGACCCACAC	58	134
		Rev	TCTCCAGTCTGCCCTTCACC		

**CX984314**	Leukocyte cell derived chemotaxin (LECT-2)	Fwd	GCGAGATGGTCAAGTTTGGTC	58	115
		Rev	TGATGCTCACGGTTTCCTCTG		

**CK991004**	Immunoglobulin heavy chain constant region (IGHC)	Fwd	AGATGGACGCTTGTGGATCTC	58	118
		Rev	GGTCTGGAGCAATGGGACAG		

**CB488287**	Secreted protein acidic and rich in cysteine precursor (SPARC)	Fwd	CCAGCAGGTCCAGGGAGTG	60	156
		Rev	TGCGTATGAGGGACTGGCTG		

**CA055453**	Glutathione peroxidase	Fwd	GCAATCAGTTCGGACATCAGG	60	131
		Rev	GTCCTTCCCATTCACATCCAC		

**CA050443**	C1q-like adipose specific protein	Fwd	GTGATGACATTTTTGAAGATCAGG	60	104
		Rev	AATAAGGAGAGAATGAAGGTGATC		

**CB516930**	CD209 antigen	Fwd	CCCATCTCCAATCCCCTTCC	60	119
		Rev	CCTGCACAGCAAGGAACAGC		

**CA051187**	Mannose-binding protein C precursor	Fwd	CAAGAGGGGCTTGGTGTTGG	60	107
		Rev	TGTTGTCAACATTGAGCCATGC		

**CA039888**	IgM heavy chain membrane bound form	Fwd	TCTGGGTTGCATTGCCACTG	60	121
		Rev	GTAGCTTCCACTGGTTTGGAC		

**CB492684**	Annexin A1	Fwd	AGGAAGGGAACAGACTGCTC	60	120
		Rev	AATAGCCTTTGCCACATCCAC		

**CB503743**	CC chemokine (CCL 19)	Fwd	CCATGTAGCAGCAAGCACAG	66	128
		Rev	GGCAGCTATCCGACATCCTC		

**CB510333**	Cd 63	Fwd	AACAGTCTGACACCCCATCTG	58	97
		Rev	TGTGCCAGACTCCTGCTGTG		

**CA056108**	C type Lectin receptor A (CTL)	Fwd	ATCCTGCACAGCAAGGAACAG	58	128
		Rev	TTGTCCACCCATCTCCAATCC		

**CK990996**	Metallothionine	Fwd	GGACAGCAGGGGCAGCAAC	58	128
		Rev	GCGATCAAAAACTGGAACATGG		

**CA058146**	Putative complement factor D	Fwd	GAATCCATCGGCTGTACGAAG	64	115
		Rev	CCGTTGGTGTGTAATGGGATG		

**AY693393**	CD8α	Fwd	CACTGAGAGAGACGGAAGACG	56	174
		Rev	TTCAAAAACCTGCCATAAAGC		

**DQ867018**	CD4	Fwd	GAGTACACCTGCGCTGTGGAAT	58	123
		Rev	GGTTGACCTCCTGACCTACAAAGG		

**FJ263446**	Interferon gamma (IFN-γ)	Fwd	CTAAAGAAGGACAACCGCAG	60	159
		Rev	CACCGTTAGAGGGAGAAATG		

**AF088999**	Inducible nitric oxide sythase (iNOS)	Fwd	GGAGAGCCTTCTGGTTG	60	116
		Rev	ACCTTAACTTGTTCCTGAGATAC		

**EF165028**	IL-10	Fwd	CGCTATGGACAGCATCCT	59	84
		Rev	AAGTGGTTGTTCTGCGTT		

**BK001401**	Arginase 1b	Fwd	CATTGGCTTGAGAGACGTGGAT	60	68
		Rev	AGTAACCTTTGACACCCAGCAA		

	Arginase 1b (Probe)		6FAM-CAGAAGAGCACCATATCC		
**AF321836**	Elongation factor-1	Fwd	GCTGTGCGTGACATGAGG	60	88
		Rev	ACTTTGTGACCTTGCCGC		

**EU418015**	GATA-3	Fwd	CCCAAGCGACGACTGTCT	60	61
		Rev	TCGTTTGACAGTTTGCACATGATG		

	GATA-3 (Probe)		FAM-TTCCTGCCCGTCTTGC		
**BT048013**	Granzyme A	Fwd	GACATCATGCTGCTGAAGTTG	60	81
		Rev	TGCCACAGGGACAGGTAACG		

**BT059144**	IL-6 receptor	Fwd	TCCCTCAGTGCTACCTCCTC	60	138
		Rev	CCAGGTGTGGCTCTCTACTC		

**GW574233**	IL-17A	Fwd	TGGTTGTGTGCTGTGTGTCTATGC	60	136
		Rev	TTTCCCTCTGATTCCTCTGTGGG		

**BT058869**	IL-17A receptor	Fwd	CAAGTGGAGGGCGATGTGTG	60	107
		Rev	TCGGGCAGGAAGAGGTTGAG		

**EU082211**	TGF-β	Fwd	AGTTGCCTTGTGATTGTGGGA	60	191
		Rev	CTCTTCAGTAGTGGTTTGTCG		

* Annealing temperature; **Product

Data analysis was done by using the LightCycler software version 4.0. The second-derivative maximum calculation was used to determine the crossing point (Cp). Since the focus was relative expression, the calibrator-normalized relative gene expression (Elongation factor 1α as the reference gene) without efficiency correction was employed for analysis. For statistical purposes, relative gene expression ratios were normalized by log transformation. Linear regression analysis was then performed with the help of STATA™ SE 9 for windows (Stata Corp. College station, TX, USA). The gene expression ratios were taken as response variables while the vaccination groups were defined as dummy variables.

To further characterize the immunological responses driving granuloma formation, the expression of additional genes not found on the microarrays including IFN-γ, IL-6 receptor, IL-10, TGF-β, IL-17A, IL-17A-receptor (IL17-AR), CD4, CD8 and iNOS was done as already described above. For arginase 1, an MGB probe (Applied Biosystems, Warrington, UK) and primers designed to anneal at the splice site were used in combination with the LightCycler^® ^Taqman^® ^Master reagents on the LightCycler instrument. A two-step PCR program (95°C for 10 sec; 60°C for 1 min) was utilized. For GATA-3, a Taqman probe in combination with LightCycler reagents as described above was used. All primer sequences are presented in Table 3.

## Authors' contributions

All authors contributed to the overall experimental design. SM and ØE designed and performed the in-vivo studies. SM, GAC and BFK carried out the microarray experiment and data analysis. SM and ISM carried out the real-time quantitative PCR. Overall analysis of the data and production of the first draft was done by SM and ØE. SM and ØE wrote the manuscript and all authors read, contributed to, and approved the final manuscript.

## Supplementary Material

Additional file 1 **Gene expression profiles by vaccine groups**. Presents a comparison of gene expression profiles between different groups of fish, categorized according to putative gene function. Click here for file

Additional file 2 **Non-immune gene differentially regulated**. Presents genes that were up or down regulated on microarrays but were classified as not linked to inflammatory or immune responses in the different vaccine groups. Click here for file

## References

[B1] Evensen Ø Brudeseth B Mutoloki S The vaccine formulation and its role in inflammatory processes in fish - effects and adverse effects Dev Biol (Basel) 2005 121 117 125 15962474

[B2] Mutoloki S Alexandersen S Evensen Ø Sequential study of antigen persistence and concomitant inflammatory reactions relative to side effects and growth of Atlantic salmon (*Salmo salar *L.) following intraperitoneal injection with oil adjuvanted vaccines Fish Shellfish Immunol 2004 16 633 645 10.1016/j.fsi.2003.10.002 15110337

[B3] Qiu B Frait KA Reich F Komuniecki E Chensue SW Chemokine expression dynamics in mycobacterial (type-1) and schistosomal (type-2) antigen-elicited pulmonary granuloma formation Am J Pathol 2001 158 1503 1515 11290568 10.1016/S0002-9440(10)64101-6 PMC1891908

[B4] Tanaka S Sato M Onitsuka T Kamata H Yokomizo Y Inflammatory cytokine gene expression in different types of granulomatous lesions during asymptomatic stages of bovine paratuberculosis Vet Pathol 2005 42 579 588 10.1354/vp.42-5-579 16145204

[B5] Constant SL Bottomly K Induction of Th1 and Th2 CD4+ T cell responses: the alternative approaches Annu Rev Immunol 1997 15 297 322 10.1146/annurev.immunol.15.1.297 9143690

[B6] Romagnani S Human Th1 and Th2 subsets: doubt no more Immunol Today 1991 12 256 257 10.1016/0167-5699(91)90120-I 1680337

[B7] Park H Li Z Yang XO Chang SH Nurieva R Wang YH A distinct lineage of CD4 T cells regulates tissue inflammation by producing interleukin 17 Nat Immunol 2005 6 1133 1141 10.1038/ni1261 16200068 PMC1618871

[B8] Infante-Duarte C Horton HF Byrne MC Kamradt T Microbial lipopeptides induce the production of IL-17 in Th cells J Immunol 2000 165 6107 6115 11086043 10.4049/jimmunol.165.11.6107

[B9] Aggarwal S Ghilardi N Xie MH de Sauvage FJ Gurney AL Interleukin-23 promotes a distinct CD4 T cell activation state characterized by the production of interleukin-17 J Biol Chem 2003 278 1910 1914 10.1074/jbc.M207577200 12417590

[B10] Krakowski M Owens T Interferon-gamma confers resistance to experimental allergic encephalomyelitis Eur J Immunol 1996 26 1641 1646 10.1002/eji.1830260735 8766573

[B11] Boniface K Blom B Liu YJ de Waal MR From interleukin-23 to T-helper 17 cells: human T-helper cell differentiation revisited Immunol Rev 2008 226 132 146 10.1111/j.1600-065X.2008.00714.x 19161421 PMC3660846

[B12] Torchinsky MB Blander JM T helper 17 cells: discovery, function, and physiological trigger Cell Mol Life Sci 2010 20054607 10.1007/s00018-009-0248-3 PMC11115816

[B13] Ogura H Murakami M Okuyama Y Tsuruoka M Kitabayashi C Kanamoto M Interleukin-17 promotes autoimmunity by triggering a positive-feedback loop via interleukin-6 induction Immunity 2008 29 628 636 10.1016/j.immuni.2008.07.018 18848474

[B14] Ivanov II McKenzie BS Zhou L Tadokoro CE Lepelley A Lafaille JJ The orphan nuclear receptor RORgammat directs the differentiation program of proinflammatory IL-17+ T helper cells Cell 2006 126 1121 1133 10.1016/j.cell.2006.07.035 16990136

[B15] Korn T Bettelli E Oukka M Kuchroo VK IL-17 and Th17 Cells Annu Rev Immunol 2009 27 485 517 10.1146/annurev.immunol.021908.132710 19132915

[B16] Bettelli E Korn T Kuchroo VK Th17: the third member of the effector T cell trilogy Curr Opin Immunol 2007 19 652 657 10.1016/j.coi.2007.07.020 17766098 PMC2288775

[B17] Langrish CL Chen Y Blumenschein WM Mattson J Basham B Sedgwick JD IL-23 drives a pathogenic T cell population that induces autoimmune inflammation J Exp Med 2005 201 233 240 10.1084/jem.20041257 15657292 PMC2212798

[B18] Aggarwal S Gurney AL IL-17: prototype member of an emerging cytokine family J Leukoc Biol 2002 71 1 8 11781375

[B19] Spriggs MK Interleukin-17 and its receptor J Clin Immunol 1997 17 366 369 10.1023/A:1027360106635 9327335

[B20] Jovanovic DV Di Battista JA Martel-Pelletier J Jolicoeur FC He Y Zhang M IL-17 stimulates the production and expression of proinflammatory cytokines, IL-beta and TNF-alpha, by human macrophages J Immunol 1998 160 3513 3521 9531313

[B21] Gaffen SL Structure and signalling in the IL-17 receptor family Nat Rev Immunol 2009 9 556 567 10.1038/nri2586 19575028 PMC2821718

[B22] Dong C TH17 cells in development: an updated view of their molecular identity and genetic programming Nat Rev Immunol 2008 8 337 348 10.1038/nri2295 18408735

[B23] Martinez GJ Nurieva RI Yang XO Dong C Regulation and function of proinflammatory TH17 cells Ann N Y Acad Sci 2008 1143 188 211 10.1196/annals.1443.021 19076351 PMC5793850

[B24] Munder M Eichmann K Moran JM Centeno F Soler G Modolell M Th1/Th2-regulated expression of arginase isoforms in murine macrophages and dendritic cells J Immunol 1999 163 3771 3777 10490974

[B25] Eisenstein TK Huang D Meissler JJ Jr al-Ramadi B Macrophage nitric oxide mediates immunosuppression in infectious inflammation Immunobiology 1994 191 493 502 7713563 10.1016/S0171-2985(11)80455-9

[B26] Nathan C Nitric oxide as a secretory product of mammalian cells FASEB J 1992 6 3051 3064 1381691

[B27] Kroncke KD Fehsel K Kolb-Bachofen V Inducible nitric oxide synthase and its product nitric oxide, a small molecule with complex biological activities Biol Chem Hoppe Seyler 1995 376 327 343 7576227 10.1515/bchm3.1995.376.6.327

[B28] MacMicking J Xie QW Nathan C Nitric oxide and macrophage function Annu Rev Immunol 1997 15 323 350 10.1146/annurev.immunol.15.1.323 9143691

[B29] Adler H Beland JL Del-Pan NC Kobzik L Brewer JP Martin TR Suppression of herpes simplex virus type 1 (HSV-1)-induced pneumonia in mice by inhibition of inducible nitric oxide synthase (iNOS, NOS2) J Exp Med 1997 185 1533 1540 10.1084/jem.185.9.1533 9151890 PMC2196291

[B30] Joerink M Savelkoul HF Wiegertjes GF Evolutionary conservation of alternative activation of macrophages: structural and functional characterization of arginase 1 and 2 in carp (*Cyprinus carpio *L.) Mol Immunol 2006 43 1116 1128 10.1016/j.molimm.2005.07.022 16257446

[B31] Secombes CJ Bird S Zou J Adaptive immunity in teleosts: cellular immunity Dev Biol (Basel) 2005 121 25 32 15962467

[B32] Zou J Carrington A Collet B Dijkstra JM Yoshiura Y Bols N Identification and Bioactivities of IFN-{gamma} in Rainbow Trout *Oncorhynchus mykiss*: The First Th1-Type Cytokine Characterized Functionally in Fish J Immunol 2005 175 2484 2494 16081820 10.4049/jimmunol.175.4.2484

[B33] Laing KJ Hardie LJ Aartsen W Grabowski PS Secombes CJ Expression of an inducible nitric oxide synthase gene in rainbow trout *Oncorhynchus mykiss* Dev Comp Immunol 1999 23 71 85 10.1016/S0145-305X(98)00036-6 10220070

[B34] Neumann NF Fagan D Belosevic M Macrophage activating factor(s) secreted by mitogen stimulated goldfish kidney leukocytes synergize with bacterial lipopolysaccharide to induce nitric oxide production in teleost macrophages Dev Comp Immunol 1995 19 473 482 10.1016/0145-305X(95)00032-O 8773198

[B35] Joerink M Forlenza M Ribeiro CM de Vries BJ Savelkoul HF Wiegertjes GF Differential macrophage polarisation during parasitic infections in common carp (Cyprinus carpio L.) Fish Shellfish Immunol 2006 21 561 571 10.1016/j.fsi.2006.03.006 16684608

[B36] Koppang EO Bjerkas I Haugarvoll E Chan EK Szabo NJ Ono N Vaccination-induced systemic autoimmunity in farmed Atlantic salmon J Immunol 2008 181 4807 4814 18802084 10.4049/jimmunol.181.7.4807

[B37] Cox JC Coulter AR Adjuvants--a classification and review of their modes of action Vaccine 1997 15 248 256 10.1016/S0264-410X(96)00183-1 9139482

[B38] Press CM Evensen Ø Reitan LJ Landsverk T Retention of furunculosis vaccine components in Atlantic salmon, *Salmo salar *L., following different routes of vaccine administration J Fish Dis 1996 19 215 224 10.1111/j.1365-2761.1996.tb00128.x

[B39] Espenes A Antigen trapping tissues in salmonid fish - with emphasis on the structure and function of splenic elipsoids 1997 Norwegian College of Veterinary Medicine

[B40] Grove S Hoie S Evensen O Distribution and retention of antigens of *Aeromonas salmonicida *in Atlantic salmon (*Salmo salar *L.) vaccinated with a DeltaaroA mutant or formalin-inactivated bacteria in oil-adjuvant Fish Shellfish Immunol 2003 15 349 358 10.1016/S1050-4648(02)00184-5 12969656

[B41] Mutoloki S Brudeseth B Reite OB Evensen O The contribution of *Aeromonas salmonicida *extracellular products to the induction of inflammation in Atlantic salmon (*Salmo salar *L.) following vaccination with oil-based vaccines Fish Shellfish Immunol 2006 20 1 11 10.1016/j.fsi.2005.01.005 16018934

[B42] Mutoloki S Reite OB Brudeseth B Tverdal A Evensen O A comparative immunopathological study of injection site reactions in salmonids following intraperitoneal injection with oil-adjuvanted vaccines Vaccine 2006 24 578 588 16221512 10.1016/j.vaccine.2005.08.070

[B43] von Schalburg KR Rise ML Cooper GA Brown GD Gibbs AR Nelson CC Fish and chips: various methodologies demonstrate utility of a 16,006-gene salmonid microarray BMC Genomics 2005 6 126 10.1186/1471-2164-6-126 16164747 PMC1239916

[B44] Purcell MK Nichols KM Winton JR Kurath G Thorgaard GH Wheeler P Comprehensive gene expression profiling following DNA vaccination of rainbow trout against infectious hematopoietic necrosis virus Mol Immunol 2006 43 2089 2106 10.1016/j.molimm.2005.12.005 16426680

[B45] Martin SA Blaney SC Houlihan DF Secombes CJ Transcriptome response following administration of a live bacterial vaccine in Atlantic salmon (*Salmo salar*) Mol Immunol 2006 43 1900 1911 10.1016/j.molimm.2005.10.007 16313960

[B46] Goetz FW Iliev DB McCauley LA Liarte CQ Tort LB Planas JV Analysis of genes isolated from lipopolysaccharide-stimulated rainbow trout (*Oncorhynchus mykiss*) macrophages Mol Immunol 2004 41 1199 1210 10.1016/j.molimm.2004.06.005 15482855

[B47] Ewart KV Belanger JC Williams J Karakach T Penny S Tsoi SC Identification of genes differentially expressed in Atlantic salmon (*Salmo salar*) in response to infection by *Aeromonas salmonicida *using cDNA microarray technology Dev Comp Immunol 2005 29 333 347 10.1016/j.dci.2004.08.004 15859237

[B48] Rahman I Oxidative stress, chromatin remodeling and gene transcription in inflammation and chronic lung diseases J Biochem Mol Biol 2003 36 95 109 12542980 10.5483/bmbrep.2003.36.1.095

[B49] Hastein T Gudding R Evensen O Bacterial vaccines for fish--an update of the current situation worldwide Dev Biol (Basel) 2005 121 55 74 15962470

[B50] Matzinger P Tolerance, danger, and the extended family Annu Rev Immunol 1994 12 991 1045 8011301 10.1146/annurev.iy.12.040194.005015

[B51] Munder M Eichmann K Modolell M Alternative metabolic states in murine macrophages reflected by the nitric oxide synthase/arginase balance: competitive regulation by CD4+ T cells correlates with Th1/Th2 phenotype J Immunol 1998 160 5347 5354 9605134

[B52] Modlin RL Th1-Th2 paradigm: insights from leprosy J Invest Dermatol 1994 102 828 832 10.1111/1523-1747.ep12381958 8006444

[B53] Billiau A Matthys P Modes of action of Freund's adjuvants in experimental models of autoimmune diseases J Leukoc Biol 2001 70 849 860 11739546

[B54] Zhang L Mia MY Zheng CL Hossain MA Yamasaki F Tokunaga O The preventive effects of incomplete Freund's adjuvant and other vehicles on the development of adjuvant-induced arthritis in Lewis rats Immunology 1999 98 267 272 10.1046/j.1365-2567.1999.00854.x 10540226 PMC2326913

[B55] Poppe TT Breck O Pathology of Atlantic salmon *Salmo salar *intraperitoneally immunised with oil adjuvanted vaccine. A case report Dis Aquat Org 1997 29 219 226 10.3354/dao029219

[B56] Reite OB Evensen Ø Mast cells in the swimbladder of Atlantic salmon *Salmo salar*: Histochemistry and responses to compound 48/80 and formalin-inactivated *Aeromonas salmonicida* Dis Aquat Org 1994 20 95 100 10.3354/dao020095

[B57] Sandler NG Mentink-Kane MM Cheever AW Wynn TA Global gene expression profiles during acute pathogen-induced pulmonary inflammation reveal divergent roles for Th1 and Th2 responses in tissue repair J Immunol 2003 171 3655 3667 14500663 10.4049/jimmunol.171.7.3655

[B58] Pagliari C Fernandes ER Guedes F Alves C Sotto MN Role of mast cells as IL10 producing cells in paracoccidioidomycosis skin lesions Mycopathologia 2006 162 331 335 10.1007/s11046-006-0069-y 17123031

[B59] Metwali A de AB Blum A Elliott D Li J Qadir K Th2-type granuloma development in acute murine schistosomiasis is only partly dependent on CD4+ T cells as the source of IL-4 Eur J Immunol 2002 32 1242 1252 10.1002/1521-4141(200205)32:5<1242::AID-IMMU1242>3.0.CO;2-7 11981811

[B60] Shen F Gaffen SL Structure-function relationships in the IL-17 receptor: implications for signal transduction and therapy Cytokine 2008 41 92 104 10.1016/j.cyto.2007.11.013 18178098 PMC2667118

[B61] Moseley TA Haudenschild DR Rose L Reddi AH Interleukin-17 family and IL-17 receptors Cytokine Growth Factor Rev 2003 14 155 174 10.1016/S1359-6101(03)00002-9 12651226

[B62] McAllister F Henry A Kreindler JL Dubin PJ Ulrich L Steele C Role of IL-17A, IL-17F, and the IL-17 receptor in regulating growth-related oncogene-alpha and granulocyte colony-stimulating factor in bronchial epithelium: implications for airway inflammation in cystic fibrosis J Immunol 2005 175 404 412 15972674 10.4049/jimmunol.175.1.404 PMC2849297

[B63] Shen F Hu Z Goswami J Gaffen SL Identification of common transcriptional regulatory elements in interleukin-17 target genes J Biol Chem 2006 281 24138 24148 10.1074/jbc.M604597200 16798734

[B64] Maitra A Shen F Hanel W Mossman K Tocker J Swart D Distinct functional motifs within the IL-17 receptor regulate signal transduction and target gene expression Proc Natl Acad Sci USA 2007 104 7506 7511 10.1073/pnas.0611589104 17456598 PMC1863505

[B65] Iyoda M Shibata T Kawaguchi M Hizawa N Yamaoka T Kokubu F IL-17A and IL-17F stimulate chemokines via MAPK pathways (ERK1/2 and p38 but not JNK) in mouse cultured mesangial cells: synergy with TNF-{alpha} and IL-1{beta} Am J Physiol Renal Physiol 2009 20042461 10.1152/ajprenal.00198.2009

[B66] Roussel L Rousseau S IL-17 primes airway epithelial cells lacking functional Cystic Fibrosis Transmembrane conductance Regulator (CFTR) to increase NOD1 responses Biochem Biophys Res Commun 2009 391 1 505 9 10.1016/j.bbrc.2009.11.088 19931506

[B67] Das SJ Ciric B Marek R Sadhukhan S Caruso ML Shafagh J Functional interleukin-17 receptor A is expressed in central nervous system glia and upregulated in experimental autoimmune encephalomyelitis J Neuroinflammation 2009 6 14 10.1186/1742-2094-6-14 19400960 PMC2689857

[B68] Iwakura Y Ishigame H The IL-23/IL-17 axis in inflammation J Clin Invest 2006 116 1218 1222 10.1172/JCI28508 16670765 PMC1451213

[B69] Fava RA Postlethwaite AE Broadley KN Davidson JM Nanney LB Lucas C Transforming growth factor beta 1 (TGF-beta 1) induced neutrophil recruitment to synovial tissues: implications for TGF-beta-driven synovial inflammation and hyperplasia J Exp Med 1991 173 121 132 10.1084/jem.173.5.1121 PMC2118851 2022923

[B70] Li MO Wan YY Sanjabi S Robertson AK Flavell RA Transforming growth factor-beta regulation of immune responses Annu Rev Immunol 2006 24 99 146 10.1146/annurev.immunol.24.021605.090737 16551245

[B71] Li MO Wan YY Flavell RA T cell-produced transforming growth factor-beta1 controls T cell tolerance and regulates Th1- and Th17-cell differentiation Immunity 2007 26 579 591 10.1016/j.immuni.2007.03.014 17481928

[B72] Faria AM Weiner HL Oral tolerance and TGF-beta-producing cells Inflamm Allergy Drug Targets 2006 5 179 190 10.2174/187152806778256034 16918481

[B73] Byon JY Ohira T Hirono I Aoki T Use of a cDNA microarray to study immunity against viral hemorrhagic septicemia (VHS) in Japanese flounder (*Paralichthys olivaceus*) following DNA vaccination Fish Shellfish Immunol 2005 18 135 147 10.1016/j.fsi.2004.06.008 15475310

[B74] Kelly KA Robinson EA Rank RG Initial route of antigen administration alters the T-cell cytokine profile produced in response to the mouse pneumonitis biovar of *Chlamydia trachomatis *following genital infection Infect Immun 1996 64 4976 4983 8945535 10.1128/iai.64.12.4976-4983.1996 PMC174477

[B75] Midtlyng PJ Reitan LJ Speilberg L Experimental studies on the efficacy and side effects of intraperitoneal vaccination of Atlantic salmon (*Salmo salar L*.) against furunculosis Fish Shellfish Immunol 1996 6 335 350 10.1006/fsim.1996.0034

[B76] Brazma A Hingamp P Quackenbush J Sherlock G Spellman P Stoeckert C Minimum information about a microarray experiment (MIAME)-toward standards for microarray data Nat Genet 2001 29 365 371 10.1038/ng1201-365 11726920

[B77] Haugland O Mercy IS Romoren K Torgersen J Evensen O Differential expression profiles and gene structure of two tumor necrosis factor-alpha variants in Atlantic salmon (*Salmo salar L*.) Mol Immunol 2007 44 1663 1674 10.1016/j.molimm.2006.08.015 17045340

