# Supplementary material for: High gene expression of inflammatory markers and IL-17A correlates with severity of injection site reactions of Atlantic salmon vaccinated with oil-adjuvanted vaccines
Source: BMC Genomics. 2010 May 27;11:336. doi: 10.1186/1471-2164-11-336 (PMC2996971; doi:10.1186/1471-2164-11-336)
Supplement: Additional file 1 — Gene expression profiles by vaccine groups. Presents a comparison of gene expression profiles between different groups of fish, categorized according to putative gene function. [file 1471-2164-11-336-S1.DOC]

Comparison of gene expression profiles between different groups of fish, categorized according to putative function. Microarray data where clones were assigned as either up or down regulated based on the basis of a ratio of at least 1.5 fold change in at least 3 of the six replicates and also a statistical p value of <0.05.

| **Group** |  |  |  |  | |  |  |
| --- | --- | --- | --- | --- | --- | --- | --- |
| **1** | **Inflammatory-related genes** |  |  |  |  | |  |
|  | **FO-2 upregulated genes** | **Fold ∆** | **FO-7 upregulated genes** | **Fold ∆** | **FO-8 upregulated genes** | | **Fold ∆** |
|  | Histidine triad nucleotide-binding protein 1 **[CA043415]** | 1.6 | Annexin A5 **[****CA059313**] | 1.7 | Anterior gradient-like protein **[CB496677]** | | 2.2 |
|  |  |  | Major vault protein **[CB517149]** | 1.5 |  | |  |
|  |  |  | Annexin A1 **[CB492684]** | 1.5 |  | |  |
|  | **FO-2 downregulated genes** |  | **FO-7 down regulated genes** |  | **FO-8 downregulated genes** | |  |
|  | NADPH oxidase flavocytochrome b **[CA048562]** | -4.3 | Cyclin-dependent kinase inhibitor 1C **[CB488506]** | -2 | BTG1 protein (B-cell translocation gene 1 protein) **[CA052366]** | | -1.6 |
|  | L-plastin **[CA044561]** | -2.3 | B-cell translocation gene 1, anti-proliferative gene **[CA056659]** | -1.5 |  | |  |
|  | Pigment epithelium-derived factor **[CB492748]** | -1.8 |  |  |  | |  |
|  | Arachidonate 5-lipoxygenase **[CA055654]** | -1.7 |  |  |  | |  |
|  | CD59-like protein 2 **[CK990487]** | -1.7 |  |  |  | |  |
|  |  |  |  |  |  | |  |
|  |  |  |  |  |  | |  |
| **2** | **Innate humoral defense** |  |  |  |  | |  |
|  | **FO-2 upregulated genes** |  | **FO-7 upregulated genes** |  | **FO-8 upregulated genes** | |  |
|  | C1q-like adipose specific protein **[CA050443]** | 1.8 | Ferritin H-3 **[CK990667]** | 1.7 | Lysozyme C, type P precursor **[CB511680]** | | 7.1 |
|  |  |  | Ferritin heavy chain **[CK990310]** | 1.6 | Lysozyme C, type P precursor **[CA054167]** | | 7 |
|  |  |  | Ferritin heavy chain **[CB503780]** | 1.5 | Lysozyme II **[CA057488]** | | 3.3 |
|  |  |  | Ferritin heavy chain **[CA052539]** | 1.5 | C1q-like adipose specific protein **[CA050443]** | | 2.1 |
|  |  |  |  |  | Secernin 1 **[DN048242]** | | 2 |
|  |  |  |  |  | Complement C3-1 **[DN048269]** | | 1.8 |
|  |  |  |  |  | Complement component C6 **[CA052383]** | | 1.5 |
|  | **FO-2 downregulated genes** |  | **FO-7 down regulated genes** |  | **FO-8 downregulated genes** | |  |
|  | Trypsin-like serine protease [CA055047] | -1.6 |  |  | Integrin beta-2 precursor (CD18) **[CB505598]** | | -1.6 |

Table 4 continued.

| **Group 3** | **Antigen recognition, processing and presentation** |  |  |  |  |  |
| --- | --- | --- | --- | --- | --- | --- |
|  | **FO-2 upregulated genes** | **Fold ∆** | **FO-7 upregulated genes** | **Fold ∆** | **FO-8 upregulated genes** | **Fold ∆** |
|  | Proteasome subunit alpha type 2 **[CA038358]** | 1.7 |  |  | H-2 class II histocompatibility antigen **[CA064221]** | 2.9 |
|  |  |  |  |  | TAP2 **[CB511230]** | 2.8 |
|  |  |  |  |  | CD209 antigen-like protein E **[CB516930]** | 2.7 |
|  |  |  |  |  | Ras-related C3 botulinum toxin substrate 2 **[CA061251]** | 2.5 |
|  |  |  |  |  | C type lectin receptor A **[CA056108]** | 2.4 |
|  |  |  |  |  | Dipeptidyl-peptidase I precursor **[CB514403]** | 2.3 |
|  |  |  |  |  | Cathepsin D precursor **[CA767935]** | 2.1 |
|  |  |  |  |  | Cathepsin D precursor **[CB502979]** | 2.1 |
|  |  |  |  |  | Proteasome subunit alpha type 4 **[CB493440]** | 2.1 |
|  |  |  |  |  | Dipeptidyl-peptidase I precursor **[CB512696]** | 2.0 |
|  |  |  |  |  | Mannose-binding protein C precursor **[CA051187]** | 2.0 |
|  |  |  |  |  | Mannose-binding protein C precursor **[CA056667]** | 1.9 |
|  |  |  |  |  | Meprin A alpha-subunit precursor **[CA048927]** | 1.9 |
|  |  |  |  |  | Dipeptidyl-peptidase I precursor **[CB510547]** | 1.8 |
|  |  |  |  |  | Meprin A alpha-subunit precursor **[CB510964]** | 1.8 |
|  |  |  |  |  | Nephrosin **[CK991012]** | 1.8 |
|  |  |  |  |  | Cathepsin Y **[CK990546]** | 1.8 |
|  |  |  |  |  | Cathepsin D precursor **[CB504794]** | 1.8 |
|  |  |  |  |  | Cathepsin D precursor **[CA043554]** | 1.7 |
|  |  |  |  |  | Lysozyme type II gene **[CA064140]** | 1.7 |
|  |  |  |  |  | Cathepsin H precursor **[CB515806]** | 1.6 |
|  |  |  |  |  | Asialoglycoprotein receptor 1 **[CB511660]** | 1.6 |
|  |  |  |  |  | MHC II invariant chain S25-7 **[CB501641]** | 1.6 |
|  |  |  |  |  | MHC II invariant chain S25-7 **[CB497250]** | 1.6 |
|  |  |  |  |  | MHC II invariant chain S25-7 **[CB502659]** | 1.6 |
|  |  |  |  |  | MHC Class I Region **[CA053328]** | 1.6 |
|  |  |  |  |  | MHC class II alpha **[CA036895]** | 1.6 |

Table 4 continued.

| **Group 3** | **Antigen recognition, processing and presentation** |  |  |  |  | |  |
| --- | --- | --- | --- | --- | --- | --- | --- |
|  | **FO-2 upregulated genes** | **Fold ∆** | **FO-7 upregulated genes** | **Fold ∆** | **FO-8 upregulated genes** | | **Fold ∆** |
|  |  |  |  |  | Proteasome subunit beta type 7 precursor **[CB511841]** | | 1.5 |
|  |  |  |  |  | FK506-binding protein 1B **[CA049957]** | | 1.6 |
|  |  |  |  |  | CD63 **[CA041450]** | | 1.6 |
|  |  |  |  |  | CD63 antigen **[CB510333]** | | 1.6 |
|  |  |  |  |  | Calreticulin **[CA041507]** | | 1.6 |
|  | **FO-2 downregulated genes** |  | **FO-7 down regulated genes** |  | **FO-8 downregulated genes** | |  |
|  | Low molecular mass protein 7 (LMP7) **[CA048585]** | -5.1 | MHC class I b region [CA057013] | -1.7 | MHC class I antigen **[CA040697]** | | -1.6 |
|  | MHC class I antigen **[CA040960]** | -3.2 | MHC class I a region **[CA051703]** | -1.6 | TAP2 **[CA051080]** | | -1.6 |
|  | MHC class I antigen **[CB503332]** | -1.8 |  |  | Proteasome subunit alpha type 5 **[CB488913]** | | -1.5 |
|  | Proteasome subunit beta type 8 precursor **[CB496486]** | -1.8 |  |  |  | |  |
|  | Transferrin gene **[CA043526]** | -1.7 |  |  |  | |  |
|  | Transferrin gene **[CB512525 ]** | -1.6 |  |  |  | |  |
|  |  |  |  |  |  | |  |
| **4** | **Cell signalling** |  |  |  |  | |  |
|  | **FO-2 upregulated genes** |  | **FO-7 upregulated genes** |  | **FO-8 upregulated genes** | |  |
|  |  |  | Hematopoietic lineage cell specific protein **[CA050621]** | 1.8 | CC chemokine **[CB503743]** | | 3.1 |
|  |  |  | Protein kinase C eta type **[CA061998]** | 1.8 | Leukocyte cell-derived chemotaxin 2 precursor **[CX984314]** | | 2.2 |
|  |  |  | Leukocyte cell-derived chemotaxin 2 precursor **[CX984314]** | 1.8 | Interferon regulatory factor 1 **[CA043655]** | | 1.9 |
|  |  |  |  |  | Syndecan-4 precursor **[CB489724]** | | 1.7 |
|  |  |  |  |  | Syntenin 1 **[CA770242}]** | | 1.6 |
|  |  |  |  |  |  | |  |
|  |  |  |  |  |  | |  |
|  | **FO-2 downregulated genes** |  | **FO-7 down regulated genes** |  | **FO-8 downregulated genes** | |  |
|  | Insulin-like growth factor I precursor **[CB506246]** | -1.6 | Insulin-like growth factor binding protein 5 **[CB514361]** | -2.6 | Regulator of G protein signalling domain [**CA051091]** | | -2.1 |
|  |  |  | follicle stimulating hormone beta subunit **[CB511858]** | -1.8 | Guanine nucleotide-binding protein G **[CB507041]** | | -2 |
|  |  |  | Guanine nucleotide-binding protein G **[CB507041]** | -1.6 | 4-3-3 protein (*Fagus sylvatica* ) **[DN048214]** | | -1.7 |
|  |  |  | inositol polyphosphate-4-phosphatase isoform **[CA046003]** | -1.6 | 4.1 protein C-terminal domain **[CA064237]** | -1.6 | |

Table 4 continued

| **Group 5** | **Immunoglobulins and T cell-related genes** |  |  |  |  |  |
| --- | --- | --- | --- | --- | --- | --- |
|  | **FO-2 upregulated genes** | **Fold ∆** | **FO-7 upregulated genes** | **Fold ∆** | **FO-8 upregulated genes** | **Fold ∆** |
|  |  |  | Immunoglobulin heavy chain **[CA051140]** | -1.7 | Immunoglobulin heavy chain constant region **[CK991004]** | 4.9 |
|  |  |  |  |  | IgM heavy chain membrane bound form **[CA039888]** | 3.8 |
|  |  |  |  |  | Immunoglobulin mu heavy chain secretory form **[CA040384]** | 3.8 |
|  |  |  |  |  | IgM heavy chain membrane bound form **[CB506793]** | 3.8 |
|  |  |  |  |  | Ig gamma-2B chain C region, membrane-bound form **[CA049564]** | 3.7 |
|  |  |  |  |  | Ig gamma-2B chain C region, membrane-bound form **[CA061887]** | 3.6 |
|  |  |  |  |  | IgM heavy chain membrane bound form **[CA049412]** | 3.5 |
|  |  |  |  |  | Ig gamma-2B chain C region, membrane-bound form [**CA048733]** | 3.5 |
|  |  |  |  |  | Immunoglobulin heavy chain constant region **[CB499462]** | 3.4 |
|  |  |  |  |  | Immunoglobulin light chain precursor **[CA050852]** | 3.0 |
|  |  |  |  |  | Immunoglobulin light chain precursor **[CA055856]** | 2.9 |
|  |  |  |  |  | IgM heavy chain membrane bound form **[CN442503]** | 2.9 |
|  |  |  |  |  | Immunoglobulin light chain precursor **[CB494545]** | 2.9 |
|  |  |  |  |  | Immunoglobulin light chain precursor **[CA057598]** | 2.9 |
|  |  |  |  |  | Immunoglobulin light chain precursor **[CA064385]** | 2.9 |
|  |  |  |  |  | Immunoglobulin light chain precursor **[CA052942]** | 2.8 |
|  |  |  |  |  | Immunoglobulin light chain precursor **[CA053420]** | 2.5 |
|  |  |  |  |  | Immunoglobulin light chain precursor **[CB516386]** | 2.5 |
|  |  |  |  |  | Immunoglobulin light chain precursor **[CN442522]** | 2.4 |
|  |  |  |  |  | Immunoglobulin light chain precursor **[CA058587]** | 2.4 |
|  |  |  |  |  | Immunoglobulin light chain variable region **[CB517114]** | 2.4 |
|  |  |  |  |  | Immunoglobulin light chain **[CA056293]** | 2.4 |
|  |  |  |  |  | Immunoglobulin light chain F class **[CB501041]** | 2.0 |
|  |  |  |  |  | Immunoglobulin light chain **[CA043397]** | 1.9 |

Table 4 continued.

| **Group 5** | **Immunoglobulins and T cell-related genes** |  |  |  |  |  |
| --- | --- | --- | --- | --- | --- | --- |
|  | **FO-2 upregulated genes** | **Fold ∆** | **FO-7 upregulated genes** | **Fold ∆** | **FO-8 upregulated genes** | **Fold ∆** |
|  |  |  |  |  | Compliment factor D **[CA058146]** | 1.9 |
|  |  |  |  |  | Immunoglobulin tau heavy chain membrane-bound form **[CA042638]** | 1.8 |
|  |  |  |  |  | Immunoglobulin tau heavy chain membrane-bound form **[CA042638]** | 1.8 |
|  |  |  |  |  | Immunoglobulin light chain **[CA062951]** | 1.8 |
|  |  |  |  |  | Immunoglobulin light chain **[CA042023]** | 1.6 |
|  |  |  |  |  | Immunoglobulin light chain **[CA056779]** | 1.5 |
|  |  |  |  |  |  |  |
|  | **FO-2 downregulated genes** |  | **FO-7 down regulated genes** |  | **FO-8 downregulated genes** |  |
|  | Tyrosine-protein kinase ZAP-70 **[CA052716]** | -1.5 |  |  | Immunoglobulin heavy chain **[CB515138]** | -3 |
|  |  |  |  |  | T-cell receptor alpha chain precursor **[CB498619]** | -2.7 |
|  |  |  |  |  | IgH.A locus **[CA061243]** | -2.4 |
|  |  |  |  |  | Signaling lymphocyte activation molecule **[CA062753]** | -2.4 |
|  |  |  |  |  | T cell receptor alpha chain **[CB510337]** | -2.2 |
|  |  |  |  |  | T cell receptor alpha chain **[CB516976]** | -2.2 |
|  |  |  |  |  | Tyrosine-protein kinase ZAP-70 **[CA052716]** | -2 |
|  |  |  |  |  | Thymus-specific serine protease precursor **[CA050763]** | -1.6 |
|  |  |  |  |  |  |  |
| **6** | **Matrix and cellular differentiation** |  |  |  |  |  |
|  | **FO-2 upregulated genes** |  | **FO-7 upregulated genes** |  | **FO-8 upregulated genes** |  |
|  | SPARC precursor **[CB488287]** | 2 |  |  |  |  |
|  | Cystatin-like domains [**CB503672]** | 1.7 |  |  |  |  |
|  | Tumor protein D53 **[CA038353]** | 1.5 |  |  |  |  |
|  | **FO-2 downregulated genes** |  | **FO-7 down regulated genes** |  | **FO-8 downregulated genes** |  |
|  |  |  |  |  | COP9 signalosome complex subunit 3 **[CA058558 ]** | -1.8 |

Table 4 continued.

| **7** | **Oxidative and antioxidative genes** |  |  |  |  | |  |
| --- | --- | --- | --- | --- | --- | --- | --- |
|  | **FO-2 upregulated genes** | **Fold ∆** | **FO-7 upregulated genes** | **Fold ∆** | **FO-8 upregulated genes** | **Fold ∆** | |
|  | Metalloproteinase inhibitor 2 precursor **[CB507385]** | 1.9 |  |  | Glutathione peroxidase-gastrointestinal **[CA055453]** | | 2.0 |
|  |  |  |  |  | Cytochrome P450 **[CA053315]** | | 1.8 |
|  |  |  |  |  | Glutathione peroxidase-gastrointestinal **[CB497633]** | | 1.7 |
|  |  |  |  |  | Metallothionein-IV **[CK990996]** | | 1.7 |
|  |  |  |  |  | Glutathione S-transferase Mu 5 **[CB497579]** | | 1.6 |
|  |  |  |  |  | Endoplasmin precursor **[CB492804]** | | 1.6 |
|  |  |  |  |  | Metallothionein-I **[CB508872]** | | 1.6 |
|  |  |  |  |  | Metallothionein-I **[CB507722]** | | 1.5 |
|  |  |  |  |  | Metallothionein-I **[CK990592]** | | 1.5 |
|  |  |  |  |  | Endoplasmin precursor **[CA769697]** | | 1.5 |
|  |  |  |  |  | Selenoprotein P precursor **[CB510644]** | | 1.5 |
|  |  |  |  |  |  | |  |
|  | **FO-2 downregulated genes** |  | **FO-7 down regulated genes** |  | **FO-8 downregulated genes** | |  |
|  |  |  | Selenoprotein P precursor **[CB498862]** | -2 | Thioredoxin **[CK990826]** | | -2.4 |
|  |  |  | Glutathione S-transferase **[CA054940]** | -1.5 | Cytochrome P450 4B1 **[CA055070]** | | -1.7 |
|  |  |  |  |  | Peroxiredoxin 1 **[CB496727]** | | -1.6 |
|  |  |  |  |  | Glutathione S-transferase **[CA057678]** | | -1.5 |
|  |  |  |  |  | Serine/threonine protein phosphatase 2A **[CK991234]** | | -1.5 |
