# Supplementary material for: High gene expression of inflammatory markers and IL-17A correlates with severity of injection site reactions of Atlantic salmon vaccinated with oil-adjuvanted vaccines
Source: BMC Genomics. 2010 May 27;11:336. doi: 10.1186/1471-2164-11-336 (PMC2996971; doi:10.1186/1471-2164-11-336)
Supplement: Additional file 2 — Non-immune gene differentially regulated. Presents genes that were up or down regulated on microarrays but were classified as not linked to inflammatory or immune responses in the different vaccine groups. [file 1471-2164-11-336-S2.DOC]

Genes that were up or down regulated on microarrays but were classified as not directly linked to inflammatory or immune responses

| **Metabolism** |  |  |  |  |  |
| --- | --- | --- | --- | --- | --- |
| **FO-2 upregulated genes** | **fold ∆** | **FO-7 upregulated genes** | **fold ∆** | **FO-8 upregulated genes** | **fold ∆** |
| NADH-ubiquinone oxidoreductase B9 subunit [**CB510353]** | 2.0 | Adenylate kinase isoenzyme 2, mitochondrial **[CA060285]** | 1.8 | Apolipoprotein B (*Salmo salar*) **[CB511166]** | 2.2 |
| Voltage-dependent anion-selective channel protein 2 **[CA055811]** | 1.7 | *Salmo salar* Na,K-ATPase alpha subunit isoform **[CA059965]** | 1.6 | Apolipoprotein E (*Oncorhynchus mykiss*) **[CB498630]** | 2.0 |
|  |  | Inorganic pyrophosphatase **[CB496548]** | 1.5 | Glucose transporter type 8 **[CB499225]** | 1.8 |
|  |  |  |  | Transaldolase **[CA060308]** | 1.7 |
|  |  |  |  | Fructose-bisphosphate aldolase A [**CB514705]** | 1.6 |
|  |  |  |  | Acyl-coenzyme A-binding protein **[CK990693]** | 1.6 |
|  |  |  |  | Transaldolase **[CA063511]** | 1.6 |
|  |  |  |  | Transaldolase (*Ctenopharyngodon idella)* **[CA063027]** | 1.6 |
|  |  |  |  | Glyceraldehyde-3-phosphate dehydrogenase (*Oncorhynchus mykiss*) **[CA042924]** | 1.6 |
|  |  |  |  | Glyceraldehyde-3-phosphate dehydrogenase II **[CA047126]** | 1.6 |
|  |  |  |  | 6-phosphogluconate dehydrogenase, decarboxylating **[CA064428]** | 1.5 |
| **FO-2 downregulated genes** |  | **FO-7 down regulated genes** |  | **FO-8 downregulated genes** |  |
| Ubiquitin-like protein SMT3C precursor **[CA049845]** | -4.0 | Guanidinoacetate N-methyltransferase **[CB494255]** | -3.1 | Ubiquitin-protein ligase E3A **[CA052560]** | -3.3 |
| Beta enolase **[CA061761]** | -1.6 | NADH-ubiquinone oxidoreductase chain 1 **[CN442557]** | -2.8 | Ubiquitin **[CA064176]** | -2.1 |
| Diamine acetyltransferase 1 **[CB488575**] | -1.6 | Cytochrome c oxidase polypeptide VIIa **[CB511353]** | -2.6 | Fructose-1,6-bisphosphatase isozyme 2 **[CB496932]** | -1.8 |
|  |  | NADH-ubiquinone oxidoreductase chain 1 **[ CA046928]** | -2.2 | NADH-ubiquinone oxidoreductase B16.6 subunit **[CB493641]** | -1.8 |
|  |  | NADH-ubiquinone oxidoreductase chain 1 **[ CN442510]** | -2.1 | 6-phosphofructokinase, liver type **[CA042530]** | -1.7 |
|  |  | NADH-ubiquinone oxidoreductase chain 1 **[ CN442551]** | -2.0 | ATP synthase D chain, mitochondrial **[CB488180]** | -1.6 |
|  |  | NADH-ubiquinone oxidoreductase chain 1 **[CK990669]** | -1.8 |  |  |
|  |  | Inositol polyphosphate-4-phosphatase **[CA046003]** | -1.6 | Kynurenine--oxoglutarate transaminase I **[CA053830]** | -1.5 |
|  |  |  |  |  |  |
|  |  |  |  |  |  |
|  |  |  |  |  |  |
|  |  |  |  |  |  |

| Appendix 1 continued |  |  |  |  |  |
| --- | --- | --- | --- | --- | --- |
|  |  |  |  |  |  |
| **Transcription/translation** |  |  |  |  |  |
| **FO-2 upregulated genes** | **fold ∆** | **FO-7 upregulated genes** | **fold ∆** | **FO-8 upregulated genes** | **fold ∆** |
| Transcription factor jun-B **[CA054491]** | 1.8 | CCAAT/enhancer binding protein beta **[CA055219]** | 1.7 | 60S ribosomal protein L10 (Oncorhynchus mykiss) **[CB497703]** | 3.6 |
| Transcription factor ETV6 **[CB494479]** | 1.8 | Transcription initiation factor TFIID subunit 10 **[CA042313]** | 1.7 | Telomerase-binding protein p23 **[CA043744]** | 2.7 |
| Eukaryotic translation initiation factor 4E binding protein 3 **[CB491023]** | 1.7 | Transcription factor jun-B **[CA056715]** | 1.6 | Heterogeneous nuclear ribonucleoprotein L **[CA064220]** | 2.2 |
| Lysyl-tRNA synthetase [*Danio rerio*] **[CB515457]** | 1.6 | 40S ribosomal protein S13 **[CB491393]** | 1.6 | DNA polymerase delta subunit 4 **[CA043782]** | 1.6 |
| Histone H1.4 **[CK991241]** | 1.5 | Interacting nucleolar phosphoprotein **[CA052388]** | 1.6 | 60S ribosomal protein L18 **[CK990468]** | 1.6 |
|  |  |  |  | RING finger protein **[CA061817]** | 1.6 |
|  |  |  |  |  |  |
| **FO-2 downregulated genes** |  | **FO-7 down regulated genes** |  | **FO-8 downregulated genes** |  |
| Putative nucleic acid binding protein **[CB516190]** | -5.4 | zinc finger protein 452 **[CA044985]** | -2.3 | Partner of Nob1 **[CA050955]** | -2.1 |
| Reverse transcriptase-like protein **[CB515142]** | -4.5 | Reverse transcriptase **[CB498751]** | -2.0 | 60S ribosomal protein L35 **[CB500108]** | -2.1 |
| Reverse Transcriptase-like protein (*Paralichthys olivaceus*) **[CA045548]** | -3.8 | Transposable element Tcb2 transposase **[CA043325]** | -1.8 | FBF1 protein **[CA063538]** | -1.9 |
| Transposase (*Pleuronectes platessa*) **[CB505847]** | -2.5 | SET protein **[CB497971]** | -1.8 | Elongation factor 1-delta **[CA056664]** | -1.9 |
| TPA: transposase **[CA051360]** | -2.4 | Tc1-like transposase **[CA042376]** | -1.7 | 40S ribosomal protein S26 **[CA061718]** | -1.8 |
| Transposase (*Pleuronectes platessa*) **[CA046627]** | -2.4 | Transposase **[CB517041]** | -1.6 | Mago nashi protein homolog **[CA051742]** | -1.7 |
| TPA: transposase (Rana pipiens) **[CA052162]** | -2.3 | Core-binding factor beta subunit **[CA051703]** | -1.6 | Elongation factor 1-delta **[CK990557]** | -1.6 |
| Putative transposase **[CK990602]** | -2.2 | Zinc-finger protein **[CB501647]** | -1.6 | 60S ribosomal protein L9 **[CK991300]** | -1.6 |
| Mouse gene for transfer RNA-Pro (CCG) [**CA047553]** | -2.1 | polymerase (RNA) III (DNA directed) polypeptide G **[CA040170]** | -1.6 | Mortality factor 4-like protein 1 **[CA050811]** | -1.6 |
| ReO_6 **[CN442511]** | -2.1 | RNA-binding protein 1 **[CA051333]** | -1.5 | Elongation factor 2 **[CA057709]** | -1.6 |
| transposase **[CB494002]** | -2.1 |  |  | Nucleolar RNA helicase II **[CA059821]** | -1.6 |
| Tc-1 like transposable element **[CA063307]** | -2.1 |  |  | Nuclear receptor coactivator 5 **[CB486790]** | -1.6 |
| Non-LTR retrotransposable element **[CA050623]** | -2.0 |  |  | Eukaryotic translation initiation factor 4E binding protein 3 **[CB510945]** | -1.5 |
| Transposase **[CK990594]** | -2.0 |  |  | 40S ribosomal protein S5 **[CB510212]** | -1.5 |
| Tripartite motif protein 39 **[CB514566]** | -1.9 |  |  |  |  |
| Transposable element Tcb2 transposase**[CA057482]** | -1.8 |  |  |  |  |
| Transposase (*Pleuronectes platessa*) [**CA047521]** | -1.8 |  |  |  |  |
| 60S ribosomal protein L35 (*Danio rerio*) **[CB500108]** | -1.7 |  |  |  |  |
|  |  |  |  |  |  |

Appendix 1 continued

| **Transcription/translation** |  |  |  |  |  |
| --- | --- | --- | --- | --- | --- |
| **FO-2 down-regulated genes** | **fold ∆** | **FO-7 down-regulated genes** | **fold ∆** | **FO-8 down-regulated genes** | **fold ∆** |
| Oncorhynchus mykiss aryl hydrocarbon receptor alpha **[CB517257]** | -1.7 |  |  |  |  |
| Transposase**[CB517566]** | -1.7 |  |  |  |  |
| TPA: transposase **[CA064431]** | -1.6 |  |  |  |  |
| Putative transposase **[CB490012]** | -1.6 |  |  |  |  |
| Transposase (*Pleuronectes platessa*) **[CB517969]** | -1.6 |  |  |  |  |
| ReO_6 **[CA059126]** | -1.5 |  |  |  |  |
| putative transposase (*Pleuronectes platessa*) **[CB490012]** | -1.5 |  |  |  |  |
| Transposase **[CB517969]** | -1.5 |  |  |  |  |
| TcA-like transposase pseudogene **[CA054202]** | -1.5 |  |  |  |  |
| ATP-dependent RNA helicase WM6 **[CB517429]** | -1.5 |  |  |  |  |
|  |  |  |  |  |  |
| **Others** |  |  |  |  |  |
| **FO-2 upregulated genes** |  | **FO-7 upregulated genes** |  | **FO-8 upregulated genes** |  |
| 23unknown |  | 12 unknown genes |  | 62 unknown |  |
| No-on-transient A protein **[CA057291]** | 2.4 | Similar to novel gene similar to D. melanogaster **[CA055675]** | 1.5 | Alpha-globin and beta-globin **[CA060492]** | 3.1 |
| Collagen alpha 1(I) chain precursor. **[CB500684]** | 2.0 | Danio rerio collagen, type I **[CB494000]** | 1.5 | Alpha-globin and beta-globin **[CA063943]** | 3.1 |
| beta-globin (Salmo salar) **[CB510950]** | 2.0 |  |  | Alpha-globin and beta-globin **[CA055246]** | 2.9 |
| Salmo salar zonadhesin-like **[CA051085]** | 1.8 |  |  | Myosin Ic **[CA042925]** | 2.8 |
| Alpha-globin and beta-globin, clone 3[**CA050663]** | 1.7 |  |  | Alpha-globin and beta-globin **[CA050663]** | 2.5 |
| PNAS-110 **[CB496558]** | 1.7 |  |  | Alpha-globin and beta-globin **[CA043223]** | 2.5 |
| Similar to DC2 protein **[CB513803]** | 1.6 |  |  | Ependymin precursor **[CA059209]** | 2.3 |
| erythrocyte band 3 protein **[CB515381]** | 1.6 |  |  | Vacuolar proton translocating ATPase **[CA062774]** | 2.1 |
| Collagen alpha 2(I) chain precursor **[CB486593]** | 1.6 |  |  | Collagen alpha 2(I) chain precursor **[CK990263]** | 2.0 |
| *Danio rerio* collagen, type I, alpha 1 **[CB498132]** | 1.6 |  |  | Similar to DC2 protein **[CB509793]** | 2.0 |
| Spectrin alpha chain **[CB493747]** | 1.6 |  |  | ependymin **[CB509787]** | 1.9 |
| Collagen alpha 1(I) chain precursor. **[CB498518]** | 1.6 |  |  | Barrier-to-autointegration factor **[CA768207]** | 1.9 |
| Collagen alpha 1(I) chain precursor. **[CB493159]** | 1.5 |  |  | PREDICTED: similar to CXorf39 protein **[CB499701]** | 1.9 |

Appendix 1 continued

|  |  |  |  |  |  |
| --- | --- | --- | --- | --- | --- |
| **Others** |  |  |  |  |  |
| **FO-2 upregulated genes** | **fold ∆** | **FO-7 upregulated genes** | **fold ∆** | **FO-8 upregulated genes** | **fold ∆** |
| Protein C14orf166 homolog. **[CB491331]** | 1.5 |  |  | Zonadhesin-like gene **[CA062112]** | 1.9 |
|  |  |  |  | Gelsolin precursor **[CA060998]** | 1.8 |
|  |  |  |  | novel gene similar to D. melanogaster CG5327 **[CA055675]** | 1.7 |
|  |  |  |  | PREDICTED: similar to DPCD protein **[CK991205]** | 1.6 |
|  |  |  |  | beta thymosin **[CB507311]** | 1.6 |
|  |  |  |  | S-100/intestinal calcium binding domain **[CB498320]** | 1.5 |
|  |  |  |  | SNF7 domain containing protein 2 **[CB517846]** | 1.5 |
|  |  |  |  | Actin-5C [CA047595]. | 1.5 |
| **FO-2 downregulated genes** |  | **FO-7 down regulated genes** |  | **FO-8 downregulated genes** |  |
| 52 unknown genes |  | 18 Unknown genes |  | 42 unknown genes |  |
| zonadhesin-like gene **[CB500101]** | -4.1 | Cyclin-dependent kinase inhibitor 1C **[CB488506]** | -2.0 | Arrdc2 protein **[CA056159]** | -2.6 |
| zonadhesin-like gene **[CA040493]** | -2.7 | Tubulin alpha-6 chain **[CB505181]** | -1.6 | Cyclin-dependent kinase 6 inhibitor **[CA041425]** | -2.4 |
| Transposase (*Pleuronectes platessa*) **[CA046627]** | -2.4 |  |  | Pulmonary surfactant-associated protein B precursor **[CB497878]** | -2.3 |
| zonadhesin-like gene **[CA042908**] | -2.3 |  |  | Aquaporin-CHIP **[CA052651]** | -2.0 |
| zonadhesin-like gene **[CB511626]** | -2.2 |  |  | Latrophilin 3 **[CA063704]** | -1.8 |
|  |  |  |  | Moesin **[CA060578]** | -1.8 |
| follicle stimulating hormone beta subunit gene **[CB491703]** | -1.9 |  |  | Phosducin-like protein 3 **[CB514417]** | -1.8 |
| follicle stimulating hormone beta subunit gene **[CA047022]** | -1.9 |  |  | Myelin basic protein **[CB497206]** | -1.7 |
| CA protein (*Schistosoma japonicum)* **[CA059348]** | -1.9 |  |  | Hemoglobin beta-2 chain **[CK990883]** | -1.7 |
| Transposase (*Pleuronectes platessa*) **[CA047521]** | -1.8 |  |  | WTNF5791 **[CA061287]** | -1.6 |
| *Salmo salar* microsatellite Rsa155 sequence **[CB508173]** | -1.7 |  |  | Circumsporozoite protein **[CB498484]** | -1.6 |
|  |  |  |  | Tropomodulin-2 **[CA051128]** | -1.6 |
|  |  |  |  | Sestrin 3 **[CB497429]** | -1.6 |
|  |  |  |  | VHSV-induced mRNA **[CA057633]** | -1.5 |
|  |  |  |  | Zonadhesin-like gene **[CA063771]** | -1.5 |
|  |  |  |  | Alveolar soft part sarcoma chromosome region, candidate 1 **[CA054110]** | -1.5 |
